# Nano Proton Scavengers Modulate Endosomal pH to Inhibit Microglial Activation and Enhance Stroke Recovery

**DOI:** 10.1002/advs.77311

**Published:** 2026-08-21

**Authors:** Xuanlin Wang, Yujing Shi, Zehua Gao, Jing Wang, Changsheng Liu

**Affiliations:** ^1^ The State Key Laboratory of Bioreactor Engineering East China University of Science and Technology Shanghai P. R. China; ^2^ School of Materials Science and Engineering Engineering Research Center for Biomedical Materials of the Ministry of Education East China University of Science and Technology Shanghai P. R. China; ^3^ Frontiers Science Center for Materiobiology and Dynamic Chemistry East China University of Science and Technology Shanghai P. R. China

**Keywords:** endosomes, inflammation, ischemic stroke, microglia, toll‐like receptors

## Abstract

Endosomes play a crucial role in immune regulation, yet their effect on microglial behavior in ischemic stroke is not well‐documented. While drug‐loaded nanoparticles can modulate microglial inflammation, their intrinsic biological effects on microglial activation are underexplored. We demonstrate that inhibiting endosomal acidification reduces pro‐inflammatory microglial polarization, limits pathological engulfment of neurons, and reduces neuronal apoptosis. To achieve the same effects in vivo, building on a validated dual‐site buffering mechanism of sulfonated chitosan, we develop sulfonated Nano Proton Scavengers (sNPS) as a materials‐based strategy to modulate endo/lysosomal pH. After cerebral ischemia, sNPS showed greater fluorescence‐associated enrichment in the ipsilateral than in the contralateral hemisphere and was associated with brain‐resident and infiltrating immune‐cell populations. In the injured brain, sNPS alleviated endo/lysosomal acid stress, suppressed TLR3/4‐linked inflammatory signaling, and normalized inflammation‐driven endo/lysosomal remodeling and proton‐loading machinery, thereby restraining maladaptive microglial activation. This immunomodulation was accompanied by improved neural structural preservation and post‐stroke survival and functional outcomes. These findings identify endosomal pH homeostasis as a tractable intracellular cue for material‐driven immunoregulation and suggest that sNPS offers a complementary therapeutic direction for ischemic stroke.

## Graphical abstract

Amphiphilic sulfonated chitosan nanoparticles buffer endo/lysosomal acidity in microglia by limiting V‐ATPase membrane recruitment and restoring endosomal homeostasis. Following systemic administration, they accumulate in the ischemic hemisphere partly through association with infiltrating immune cells, suppress TLR3/IRF3 and TLR4/NF‐κB signaling, reshape the inflammatory microenvironment, and promote neural repair and functional recovery after stroke.

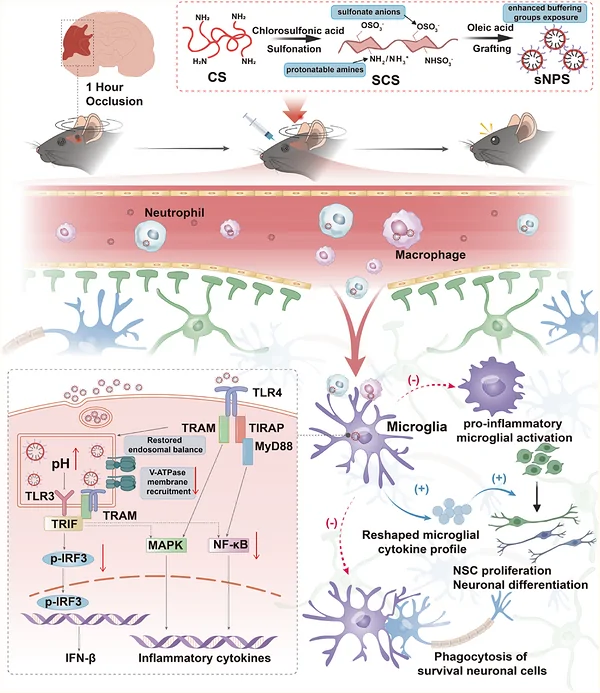

## 1 Introduction

Ischemic stroke is a leading cause of death and disability worldwide, with a higher incidence in the elderly population [1, 2, 3]. The cornerstone of ischemic stroke treatment is the rapid restoration of blood flow to the ischemic region through intravenous thrombolysis with recombinant tissue plasminogen activator (rt‐PA) within 4.5 h and mechanical thrombectomy within 6–24 h [4, 5, 6]; however, reperfusion can also cause secondary tissue damage by initiating an inflammatory storm that recruits peripheral cells to infiltrate brain tissue, releasing more destructive reactive oxygen species and pro‐inflammatory cytokines [7]. To prevent neuronal loss caused by reperfusion injury, neuroprotective agents such as edaravone and *N*‐butylphthalide are used clinically to reduce neuronal apoptosis, but their limited specificity and the complex pathological environment of stroke have led to unsatisfactory therapeutic outcomes [8]. Accordingly, there remains a critical demand for the development of alternative interventions that address broader therapeutic targets beyond neuronal preservation.

Microglia, the primary immune cells within the central nervous system (CNS), play a crucial role in monitoring and responding to pathological changes [9, 10], with their involvement in immune modulation processes significantly affecting stroke pathology and prognosis. During ischemic stroke, insufficient oxygen and glucose supply rapidly activates microglia to initiate and regulate local inflammatory responses by releasing various cytokines (such as IL‐1β [11], TNF‐α [11, 12], and IL‐6 [13]), chemokines, and other mediators. The activation process is accompanied by morphological changes; specifically, resting microglia (with long processes) undergo process retraction and gradually transform into amoeboid‐like cells (with short and thick processes) [14, 15, 16]. Microglial activation can have protective effects by facilitating the clearance of dead cells and debris, thereby promoting tissue repair [17]. However, excessive or prolonged activation of microglia may exacerbate brain tissue damage and increase infarct size by phagocytosing normal neuronal cells [18]. Upon activation, microglia are traditionally asserted transforming into pro‐inflammatory M1 phenotypes and anti‐inflammatory M2 phenotypes, thereby either exacerbating or ameliorating the tissue repair following ischemic stroke [19, 20, 21, 22]. However, the widespread application of sequencing in recent years has led to the identification of distinct microglial subtypes in various neurodegenerative diseases, while modulating the proportions of these specific subtypes can directly impact the pathological process [23, 24, 25]. Thus, regulation of microglial heterogeneity or activation offers effective means to treat ischemic stroke.

Endosomes, as critical organelles responsible for transport and signal transduction, play a significant role in the immunoregulatory processes of various neurodegenerative diseases [26, 27]. Endosome‐mediated Toll‐like receptor (TLR) signaling pathways directly determine the fate of various immune cells. In macrophages, endosome‐mediated TLR3 activation triggers NF‐κB via downstream TRAF6, thereby inducing the secretion of inflammation‐related factors [28]. For plasmacytoid dendritic cells (pDCs), the activation of TLR7 and TLR9 signals on endosomes recruits MyD88, which interacts with IRAK‐1, leading to the phosphorylation and activation of IRF7, and subsequently, the production of type I interferons (IFN) [29]. Microglia, as the “vanguard” in the immune response processes of neurodegenerative diseases, also have their activation and phenotypic polarization controlled by TLR3/TLR4. Previous studies have shown that activation of the TLR4 signaling pathway directly causes secondary inflammatory damage in intracerebral hemorrhage, while antagonizing the TLR4 pathway can inhibit microglial polarization toward the M1 phenotype [30]. Furthermore, activation of the TLR3 pathway by polyinosinic:polycytidylic acid (poly I:C), an endosome‐mediated TLR3 agonist, can induce an increased proportion of activated microglia [31, 32]. Based on these findings, we hypothesize that modulating the acidic environment of endosomes through the physicochemical properties of materials may regulate microglial behavior after ischemic stroke via TLR3/4‐mediated pathways.

In this study, inhibition of endosomal acidification was demonstrated to ameliorate the pro‐inflammatory phenotype polarization of microglia, attenuate excessive phagocytic behavior, and reduce the induction of neuronal apoptosis under inflammatory conditions. Building on this mechanistic premise, a cargo‐free materials strategy was developed based on sulfonated chitosan buffering chemistry and engineered into sulfonated Nano Proton Scavengers (sNPS) (Figure 1). By concentrating proton‐buffering functionalities within endo/lysosomes and exhibiting ischemia‐associated ipsilateral enrichment, sNPS modulates endo/lysosomal pH homeostasis, dampens TLR3/4‐linked inflammatory signaling and alleviates endo/lysosomal acid stress to rebalance innate immune signaling. In the tMCAO model, this immunomodulatory effect was accompanied by restrained maladaptive microglial responses, improved neural structural preservation, and enhanced post‐stroke survival and functional outcomes. Overall, this work highlights a materials‐intrinsic strategy to regulate the post‐stroke inflammatory microenvironment by targeting endosomal pH, offering a complementary therapeutic direction for ischemia–reperfusion injury.

**FIGURE 1:**
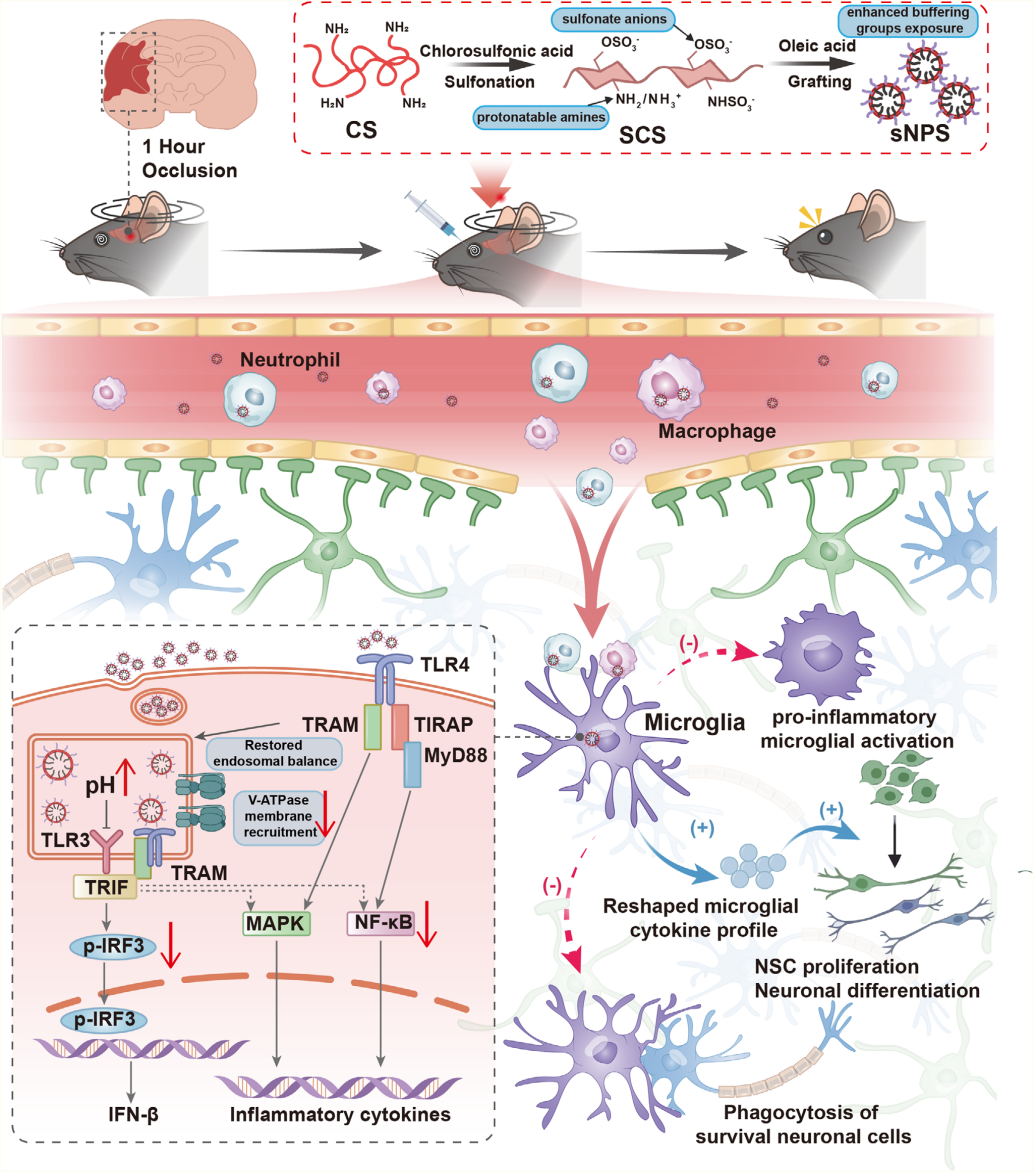
Schematic illustration of the design rationale and therapeutic mechanism of sulfonated Nano Proton Scavengers (sNPS) for ischemic stroke. Sulfonated chitosan (SCS) was generated from chitosan (CS) by chlorosulfonic acid–mediated sulfonation and served as the buffering scaffold for subsequent nanoparticle construction. The buffering behavior of SCS arises from the combined contribution of fixed sulfonate anions and protonatable amino groups, and oleic acid grafting further drives amphiphilic self‐assembly into sNPS, increasing the effective surface exposure of these buffering functionalities. After systemic administration, sNPS preferentially accumulates in the ischemic hemisphere, in part through association with infiltrating peripheral immune cells such as neutrophils and macrophages. Upon uptake by microglia, sNPS alleviates endo/lysosomal acid stress, limits V‐ATPase membrane recruitment, and helps restore endosomal balance, thereby dampening TLR3/IRF3‐ and TLR4/NF‐κB‐linked inflammatory signaling. This regulation restrains maladaptive microglial activation and reshapes the microglial cytokine profile, creating a more permissive microenvironment for neural stem cell (NSC) proliferation and neuronal differentiation, while reducing pathological microglia–neuron interaction in the ischemic brain. Together, these effects promote post‐stroke neural repair and functional recovery.

## 2 Results

### 2.1 Inhibition of Endosomal Acidification Improves the Microglial Inflammatory Response

To investigate the regulatory role of endosomal acidity on microglial behavior in the context of ischemic stroke, BV2 cells (a mouse‐derived microglial cell line) were activated using lipopolysaccharide (LPS) to mimic a pathological inflammatory environment. Two classic endosomal acidification inhibitors, bafilomycin A1 (BFA1) and chloroquine (CHQ), were then applied to treat the cells. Both treatments reduced the expression of the pro‐inflammatory genes TNF‐α, IL‐6, and IL‐1β, while producing comparatively limited effects on IL‐4 and IL‐10. Consistently, immunofluorescence analysis showed reduced IL‐6 signals but only modest changes in Arg‐1 after BFA1 or CHQ treatment (Figure 2A–C). The preferential inhibition of the pro‐inflammatory phenotype might be related to endosome‐mediated Toll‐like receptor signaling, which regulates the expression of pro‐inflammatory factors through NF‐κB activation [33, 34].

**FIGURE 2:**
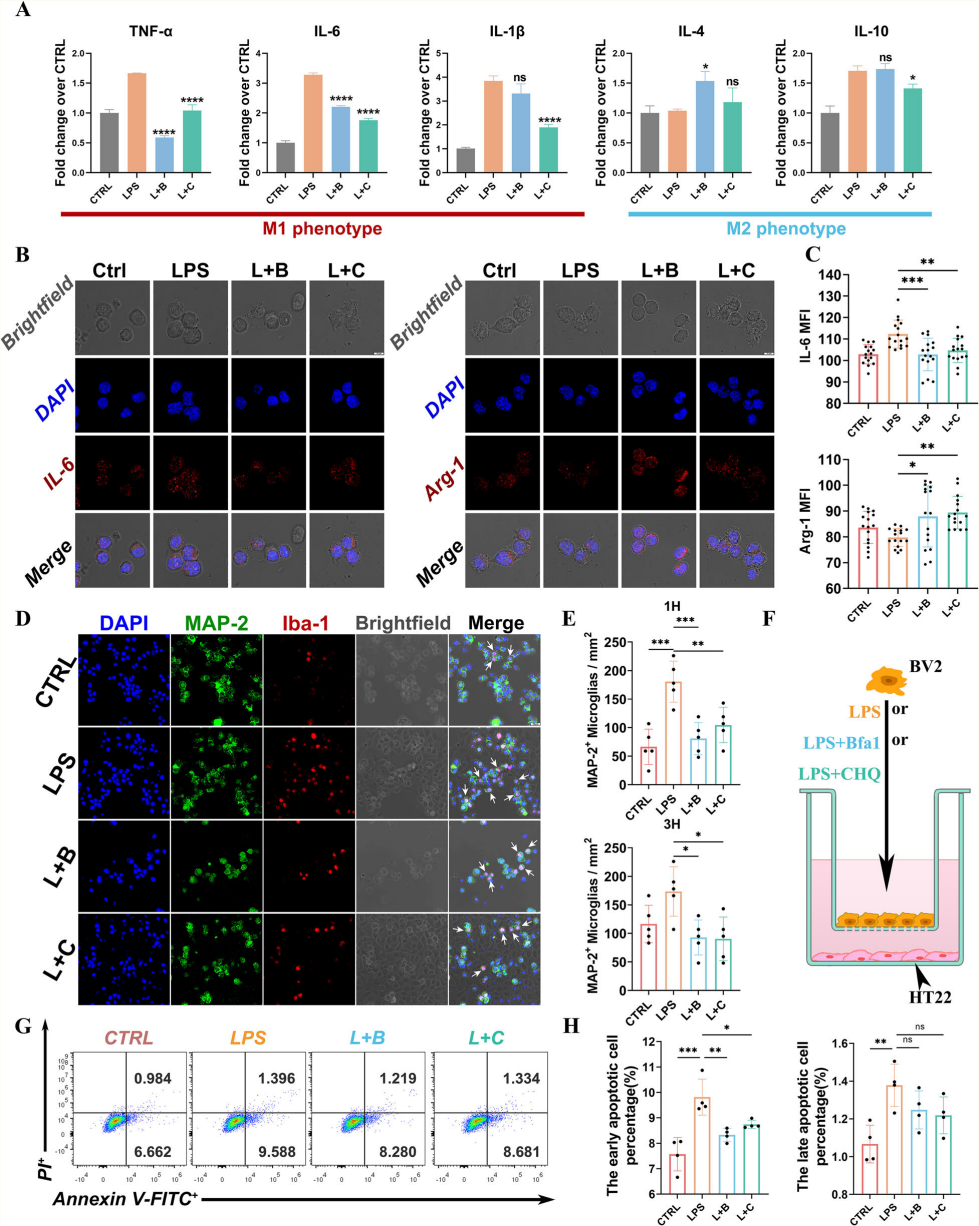
The immunoregulatory functions of endosomes in microglia. A. The expression of genes related to inflammation in BV2 cells was quantified using qRT‐PCR, with the primer sequences provided in Table S2. Data were shown as mean ± SD (n = 3). B‐C. Representative immunofluorescence images (B) and quantitative results (C) of IL‐6 and Arg‐1 expression in BV2 cells. Scale bar was 10 µm. Data were presented as mean ± SD (n = 16 cells). D‐E. Representative immunofluorescence images of HT22 cells phagocytosed by BV2 cells after 1 h co‐culture in an inflammatory environment (D) and the quantitative results of MAP‐2^+^ BV2 cells (E). BV2 cells phagocytosing HT22 cells were marked with white arrows in the figure. Scale bar was 30 µm. Data were presented as mean ± SD (n = 5). F. Schematic Diagram of the Transwell Experiment. G‐H. Representative flow cytometry plots (G) with quantification (H) of apoptosis in HT22 cells in the basal chamber. Data were presented as mean ± SD (n = 4). L + B: LPS (1000 ng mL^−1^) and BFA1 (1 µM) treatment; L+C: LPS (1000 ng mL^−1^) and CHQ (20 µM) treatment. *p < 0.05, **p < 0.01, ***p < 0.001, ****p < 0.0001.

Because excessive microglial activation can promote the engulfment of surviving or stressed neurons after ischemic stroke [35, 36, 37], we next evaluated microglia‐neuron interactions in a BV2/HT22 coculture model. LPS increased MAP‐2‐associated signals within BV2 cells, whereas BFA1 and CHQ reduced this response at both 1 and 3 h (Figure 2D,E), indicating attenuation of abnormal microglial engulfment.

After ischemic stroke, damaged neurons release a variety of signaling molecules (such as glutamate, reactive oxygen species, and damage‐associated molecular patterns (DAMP)) that directly activate the microglial cells [38, 39, 40]. The pro‐inflammatory factors secreted by activated microglial cells (such as IL‐6) [41] accelerate neuronal apoptosis, thus creating a vicious cycle. Based on the confirmed regulatory effects of BFA1 and CHQ on the pro‐inflammatory polarization of microglial cells, a transwell experiment was designed to explore whether inhibition of endosomal acidification could ameliorate neuron apoptosis induced by microglia following ischemic stroke (Figure 2F–H). Quantitative results from flow cytometry showed that LPS‐activated BV2 cells significantly induced apoptosis in HT22 cells in the basal chamber, consistent with previous reports [42]. BFA1 and CHQ intervention reduced the proportion of early neuronal apoptosis, indicating that they may confer neuroprotective effects by inhibiting the polarization of microglia toward a pro‐inflammatory phenotype (Figure 2G, H). The more evident change in early apoptosis may be related to the relatively short 3 h coculture period, which primarily captured early apoptotic responses before substantial progression to late apoptosis. These results suggest that modulating endosomal acidity could be a potential strategy for altering the immune behavior of microglia in an injury‐induced inflammatory environment like ischemic stroke.

### 2.2 Dual‐Site pH‐Buffering Basis of Sulfonated Chitosan and Construction of sNPS

Although endosomal acidification inhibitors have been clinically applied in some autoimmune diseases, such as rheumatoid arthritis [43] and systemic lupus erythematosus [44], their therapeutic effects on ischemic stroke remain scarcely reported. Additionally, the clinical use of CHQ can lead to side effects like retinopathy or seizures [45]. Comparatively, the biological effects of materials have been widely reported in recent years [46, 47]. By designing the physicochemical properties of materials, it is possible to achieve not only effective therapeutic outcomes but also enhanced biocompatibility. Therefore, we propose the innovative approach of using Nano Proton Scavengers to mimic the immunomodulatory functions of BFA1 and CHQ in microglial cells.

To regulate proton levels within endosomes, sulfonated chitosan (SCS) was chosen as the base polymer, given the coexistence of strongly anionic sulfonate groups and protonatable amino groups that could jointly contribute to pH buffering. To validate this structure–function rationale and identify the dominant chemical contributors, we synthesized a panel of chitosan derivatives with systematic variations in sulfonate density and amino‐group accessibility, including SCS with different sulfonation levels and SCS derivatives with modulated amino‐group density generated by PEGylation or arginine grafting (SP and ASCS/ACS; LA denotes arginine) (Figure 3A; Figure S2A,B). Elemental sulfur analysis confirmed distinct sulfonation degrees (SCS‐LS, low sulfonation; SCS‐HS, high sulfonation) (Figure 3B), while free‐amino‐content measurements across the derivative set revealed corresponding differences in accessible amines (Figure 3C). Consistently, acid–base titration profiles showed clear derivative‐dependent buffering capacities (Figure 3D). Notably, although high‐sulfonation SCS (SCS‐HS) contained fewer free primary amines than low‐sulfonation SCS (SCS‐LS), it exhibited stronger buffering capacity, highlighting the critical contribution of sulfonate groups; meanwhile, PEG‐ or arginine‐modified derivatives did not outperform SCS‐HS, further indicating that amino‐group accessibility also remained important. Together, these results support that the buffering behavior of SCS arises from the combined effects of sulfonate‐driven ion partitioning (Donnan‐type effect) and amine protonation/deprotonation, and therefore SCS‐HS was selected as the precursor material for subsequent sNPS construction.

**FIGURE 3:**
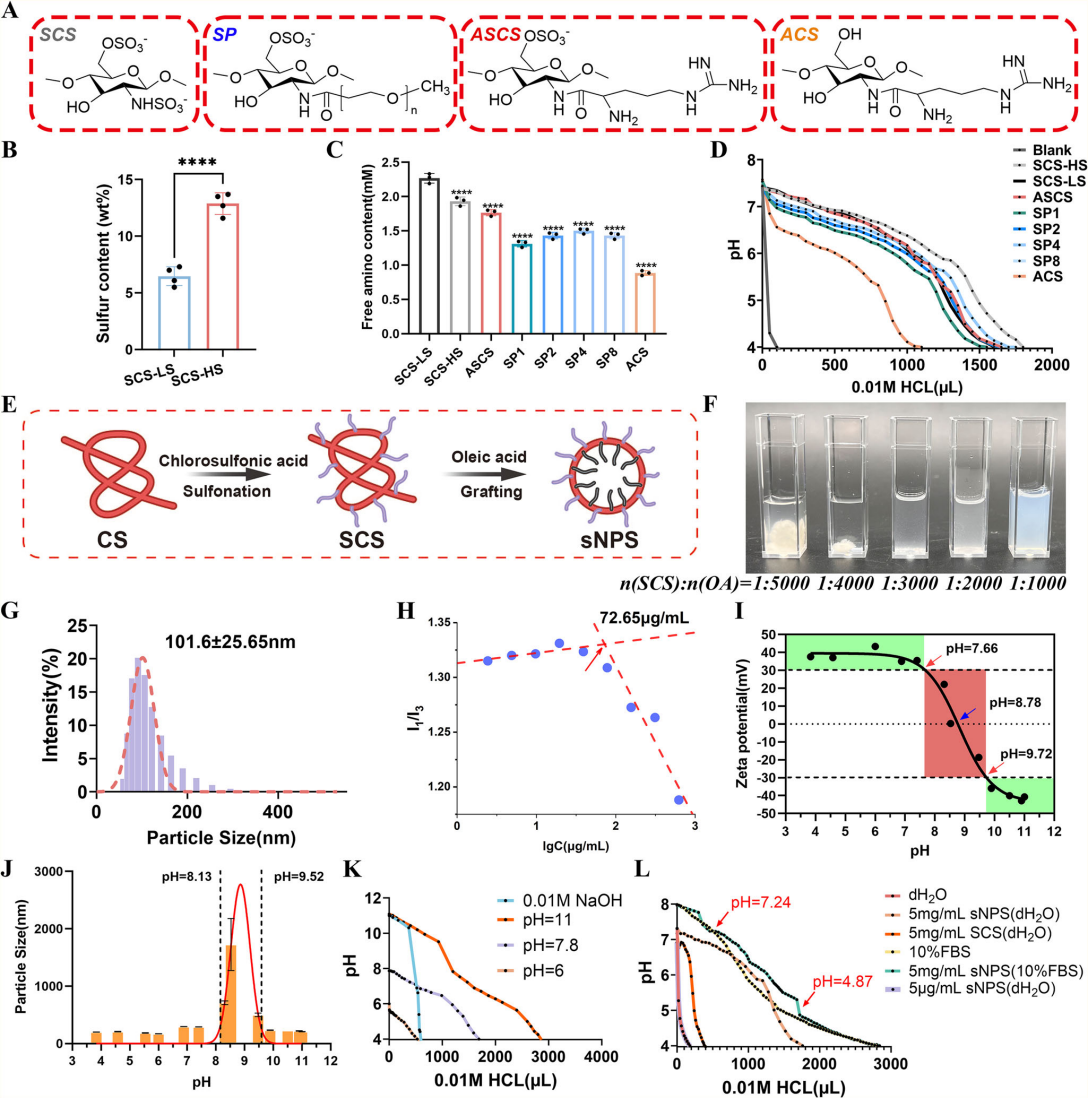
The dual‐site pH buffering mechanism of sulfonated chitosan and structural and physicochemical characterization of sNPS. A. Chemical structures of the chitosan derivatives, including sulfonated chitosan (SCS), PEG‐modified sulfonated chitosan (SP), arginine‐grafted sulfonated chitosan (ASCS), and arginine‐grafted chitosan (ACS). B. Quantification of sulfur content (wt%) in SCS synthesized at different sulfonation degrees (SCS‐LS and SCS‐HS). C. Free amino content of the indicated chitosan derivatives (SCS‐LS, SCS‐HS, ASCS, SP series, and ACS). D. Acid–base titration curves of the different derivatives (0.01 M HCl), reflecting their proton‐buffering capacities. E. Schematic illustration of sNPS fabrication. F. Representative photographs of sNPS dispersions prepared with different feed ratios of SCS and OA (*n*(SCS):*n*(OA)). G. DLS size distribution of sNPS in aqueous solution. H. Determination of the critical micelle concentration (CMC) using the pyrene fluorescence method; the inflection point in the *I*1/*I*3–logC plot is used to calculate the CMC (red arrow). I. Zeta potential of sNPS as a function of pH to determine the isoelectric point (IEP); regions with |zeta potential| > 30 mV are highlighted in green, while |zeta potential| < 30 mV are highlighted in red. J. pH‐dependent changes in hydrodynamic diameter of sNPS, with the critical pH range for size transition indicated. K. Acid titration curves of sNPS starting from different initial pH values. L. Comparison of titration behaviors of sNPS and SCS at different concentrations in dH_2_O or 10% FBS, indicating the influence of protein‐containing media on proton scavenging. Data are presented as mean ± SD. *p < 0.05, **p < 0.01, ***p < 0.001, ****p < 0.0001.

Building on SCS as the functional blueprint, we next introduced oleic acid (OA) as a hydrophobic segment to drive amphiphilic self‐assembly into sulfonated Nano Proton Scavengers (sNPS), which not only enhances the accessible presentation of sulfonate and amino groups but also converts SCS from a random‐coil macromolecule into nanoparticles that typically exhibit superior brain‐targeting/brain‐accumulation performance (Figure 3E). Nanoparticle formation was governed by the hydrophilic/hydrophobic feed ratio, and stable dispersions were obtained upon tuning the molar ratio of SCS to OA (Figure 3F). The chemical modifications were confirmed by Fourier‐transform infrared spectroscopy (FT‐IR). Compared with chitosan, SCS exhibited characteristic sulfonate‐associated absorption bands at 1258 cm^−1^ (S═O stretching) and 813 cm^−1^ (S─O stretching), indicating successful introduction of sulfonic acid groups (Figure S2C). Moreover, relative to SCS, sNPS showed additional alkyl‐related peaks at 1462 cm^−1^, 2854 cm^−1^ (─CH_3_ stretching), and 2926 cm^−1^ (─CH_2_─ stretching), consistent with successful grafting of oleic acid onto the polymer backbone (Figure S2D). The resulting sNPS exhibited a hydrodynamic size centered at 101.6 ± 25.65 nm (Figure 3G) and a critical micelle concentration of 72.65 µg mL^−1^ (Figure 3H), which facilitates the penetration of the blood‐brain barrier [48]. Transmission electron microscopy (TEM) further confirmed well‐dispersed nanoscale particles (Figure S2E; scale bar: 500 nm).

The isoelectric point is a key parameter governing colloidal stability and interfacial interactions [49]. The isoelectric point of sNPS was determined to be ∼pH 8.78 (Figure 3I). Notably, at pH values below 7.66, the absolute zeta potential exceeded 30 mV, indicating that sNPS remain colloidally stable under both physiological pH (7.4) and stroke‐relevant acidic conditions (pH 6.0–6.8). Consistently, pH‐dependent size measurements showed that the hydrodynamic diameter of sNPS remained stable at pH values below 8.13 or above 9.52, with the critical transition points indicated in Figure 3J.

Given the surface‐presented sulfonate groups and residual protonatable moieties, we next evaluated the proton‐buffering behavior of sNPS by acid–base titration. sNPS exhibited appreciable buffering capacity across both acidic and basic ranges when titration was initiated from different starting pH values (Figure 3K). In comparison, SCS also displayed buffering capacity but was consistently weaker than sNPS, which may relate to their different interfacial charge states (Figure 3L; Figure S2F). Besides, the buffering effect was concentration dependent: 5 µg mL^−1^ sNPS showed minimal buffering, whereas higher concentrations produced a pronounced buffering response (Figure 3L). Importantly, in 10% FBS, sNPS still displayed measurable buffering between pH 7.24 and 4.87, although the apparent buffering profile was attenuated relative to that in dH_2_O, likely reflecting the intrinsic buffering capacity and ionic/protein components of serum (Figure 3L). Finally, CCK‐8 assays showed no significant cytotoxicity of sNPS in BV2 cells across tested concentrations after 1 day and 3 days of incubation (Figure S2G).

### 2.3 The Accumulation of sNPS in the Ipsilateral Hemisphere of Ischemia in Vivo

Nanoparticles can be phagocytized by various peripheral inflammatory cells and, following ischemic stroke, the inflammatory storm in the infarct area can lead to the recruitment of immune cells from the peripheral blood [50, 51]. Therefore, we investigated whether sNPS exhibits enhanced accumulation and immune‐cell association in the infarcted hemisphere compared to SCS. To examine this possibility, a mouse transient middle cerebral artery occlusion (tMCAO) model was established to simulate cerebral artery embolism in clinical patients. The reduction in blood flow following the occlusion of the suture and the subsequent restoration of blood flow were observed using laser speckle contrast imaging, indicating the successful establishment of the tMCAO model (Figure S3A). Before biodistribution analysis, FITC labeling was validated by confirming efficient removal of free dye, comparable labeling efficiencies between SCS and sNPS, minimal changes in particle size and zeta potential, and stable fluorescence retention under serum‐containing conditions (Figure S3B–D).

After systemic administration, the brain distribution of FITC‐labeled SCS and sNPS was first evaluated by flow cytometry using the gating strategy shown in Figure 4A. Quantitative analysis showed that free SCS exhibited no obvious hemispheric preference, whereas sNPS displayed progressively greater enrichment in the ipsilateral hemisphere at 12 and 24 h (Figure 4B,C). No corresponding increase was observed in the contralateral hemisphere (Figure S3E). Further analysis of the FITC^+^ cell composition showed that microglia, neutrophils, and macrophages constituted the major labeled cell populations in the brain, whereas other cell types accounted for a relatively smaller fraction (Figure 4D). Together, these data demonstrate that sNPS preferentially accumulates in the ischemic hemisphere and is mainly associated with brain‐resident and infiltrating immune cells, supporting the potential involvement of these immune‐cell populations in its ischemic distribution.

**FIGURE 4:**
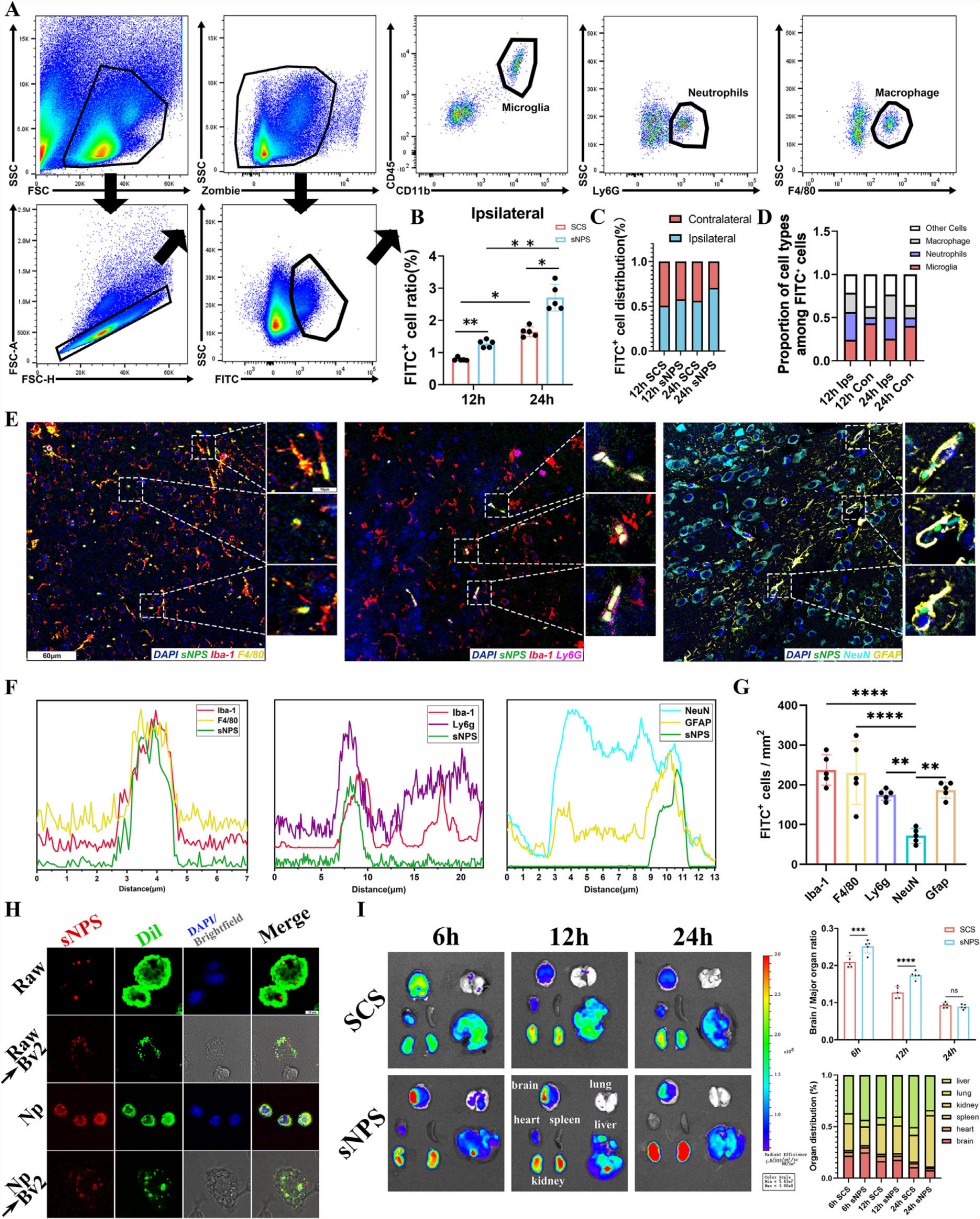
Ischemia‐associated brain accumulation and cellular distribution of sNPS after ischemic stroke. A–C. Representative flow cytometry gating strategy (A) and quantitative analysis (B, C) of fluorescently labeled sNPS/SCS‐positive (FITC^+^) cells in the ischemic contralateral and ipsilateral hemispheres at 12 h and 24 h post‐injection. Data are presented as mean ± SD (n = 5). D. Proportion of immune cell subtype (microglia, neutrophils, macrophages, and other cells) among total FITC^+^ cells in the brain at the indicated time points. E–G. Representative immunofluorescence images showing the cellular uptake of sNPS in the infarcted hemisphere (E), line‐scan colocalization profiles of sNPS fluorescence with indicated cellular markers in the magnified regions (F), and quantification of FITC^+^ phagocytic cells for each marker (G). Scale bars: 60 µm (overview) and 10 µm (magnified). Data are presented as mean ± SD (n = 5). H. Representative confocal images acquired after coculture of BV2 microglia with DiI‐labeled primary neutrophils or RAW264.7 macrophages preloaded with FITC‐sNPS, showing the distribution and spatial coexistence of FITC‐sNPS‐associated and donor‐cell‐associated DiI fluorescence. Blue, nuclei (DAPI); green, DiI‐labeled cell membrane; red, sNPS. Scale bar: 10 µm. I. Ex vivo fluorescence imaging and quantitative biodistribution of sNPS and SCS in major organs at 6 h, 12 h, and 24 h after intravenous administration in tMCAO mice. Data are presented as mean ± SD. ns, not significant; *p < 0.05, **p < 0.01, ***p < 0.001, ****p < 0.0001.

This ischemia‐associated enrichment was further supported by ex vivo imaging. In sham‐operated mice, no obvious hemispheric preference was observed at either 12 or 24 h, whereas in tMCAO mice, sNPS showed a clear preference for the ischemic hemisphere together with prolonged retention over time (Figure S3F). These findings suggest that ischemia‐associated pathological changes, including the inflammatory recruitment of peripheral immune cells, contribute to the selective accumulation of sNPS in the injured brain [52]. To further evaluate whether BBB integrity affects the passage of sNPS, an in vitro Transwell BBB coculture model was established using bEnd.3 endothelial cells and C8‐D1A astrocytes (Figure S3G). Barrier formation was first functionally evaluated using 10 kDa FITC‐dextran. FITC‐dextran permeability progressively decreased during coculture and reached a relatively stable low level by day 5, supporting the formation of a restrictive barrier; therefore, day 5 was selected for subsequent permeability experiments. FITC‐sNPS showed low leakage across the normal in vitro BBB model, whereas OGD progressively increased its passage. These results indicate that BBB injury facilitates sNPS passage across the barrier model, while the low leakage observed under the normal condition suggests that sNPS permeability is strongly influenced by barrier integrity.

To further define the cellular distribution of sNPS within the ischemic hemisphere, FITC‐labeled sNPS was administered in tMCAO mice, followed by immunofluorescence staining of the corresponding cell populations in the infarct region (Figure 4E). Colocalization and quantitative analyses together showed that sNPS was predominantly associated with F4/80^+^ macrophages, Ly6G^+^ neutrophils, and Iba‐1^+^ microglial cells, whereas its overlap with NeuN^+^ neurons was markedly lower. Among resident brain cells, sNPS signals were also observed in spatial proximity to GFAP^+^ astrocytic structures (Figure 4F,G).

In vitro coculture experiments were then performed to examine whether coculture with sNPS‐loaded peripheral immune cells could result in the acquisition of sNPS‐associated signals by brain‐resident microglia. DiI‐labeled peripheral immune cells preloaded with FITC‐sNPS were cocultured with BV2 cells (Figure 4H). Following coculture, FITC‐sNPS‐associated fluorescence and donor‐cell‐associated DiI signals were observed in cells morphologically identified as BV2 microglia based on their larger spread area and characteristic morphology, supporting redistribution of sNPS‐associated material and donor‐cell components during myeloid cell‐microglia interactions. Together with the in vivo enrichment of sNPS in F4/80^+^ macrophages and Ly6G^+^ neutrophils, these findings support the contribution of peripheral myeloid cells to the delivery and subsequent intercellular redistribution of sNPS in the ischemic brain.

Ex vivo organ imaging showed prominent hepatic and renal fluorescence‐associated distribution of both SCS and sNPS, while sNPS exhibited greater brain enrichment than SCS during the early post‐injection period (Figure 4I). These data indicate that sNPS achieves more efficient brain enrichment than SCS during the early post‐injection phase while both materials are gradually cleared through the liver–kidney axis. Despite this systemic distribution, H&E staining of the heart, liver, spleen, lung, and kidney at 48 h showed no obvious tissue injury or inflammatory infiltration after sNPS administration (Figure S3H), indicating acceptable in vivo biocompatibility.

### 2.4 sNPS Alleviates Endo/Lysosomal Acid Stress and Restrains Maladaptive Microglial Responses After Ischemic Stroke

To assess whether sNPS modulates endo/lysosomal acidity under inflammatory conditions, overall dextran uptake was monitored by FITC‐dextran, whereas pHrodo‐dextran was used as an indicator of acidic endo/lysosomal compartments (Figure 5A,B). LPS stimulation decreased the FITC‐dextran/pHrodo ratio, whereas SCS produced only a limited restorative effect and sNPS markedly increased the ratio. BFA1 produced a similar response, while combined BFA1 and sNPS treatment showed the strongest effect. This result suggests that pharmacological blockade of endosomal acidification and material‐based proton buffering may act in a partially complementary manner: while BFA1 directly interferes with the acidification machinery, sNPS may further buffer residual luminal protons after intracellular uptake, thereby producing a more pronounced suppression of endo/lysosomal acid stress. Because pHrodo fluorescence is sensitive to acidic vesicular environments and may potentially be affected by fixation, we further performed pHrodo‐dextran imaging under unfixed conditions. The unfixed‐cell images and quantitative analysis showed a trend consistent with that obtained from the fixed‐cell assay (Figure S4D,E). In addition, TEM observation did not reveal obvious endo/lysosomal swelling, membrane rupture, or cytoplasmic leakage‐like structures after sNPS treatment (Figure S4F), suggesting that the buffering effect of sNPS was not accompanied by apparent proton‐sponge‐like endosomal disruption.

**FIGURE 5:**
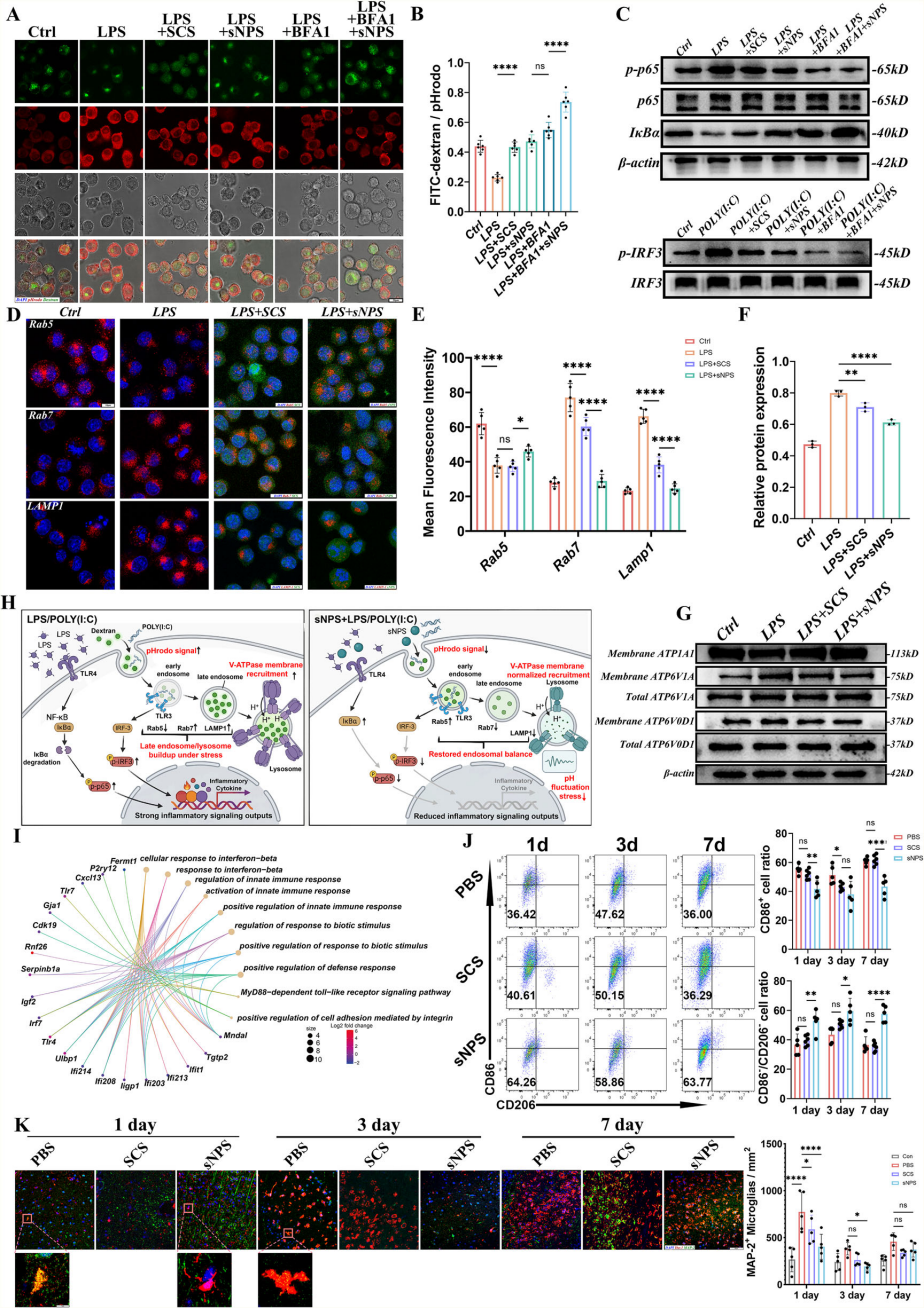
sNPS alleviates endo/lysosomal acid stress to rebalance innate immune signaling and restrains maladaptive microglial responses after ischemic stroke. A–B. Representative confocal images (A) and quantification (B) of intracellular pHrodo red–dextran (endo/lysosomal acidity) and FITC–dextran (dextran uptake/phagocytosis) in primary microglial cells under the indicated treatments. C. Western blotting analysis of key innate immune signaling proteins in primary microglia stimulated with LPS or poly(I:C) and subjected to the indicated treatments; the primary antibodies used are listed in Table S3. D–E. Representative immunofluorescence images of endosomal/lysosomal markers Rab5, Rab7, and LAMP1 (D) with mean fluorescence intensity quantification (E) in microglial cells across groups. F–G. Analysis of V‐ATPase membrane recruitment in microglial cells treated with SCS or sNPS under LPS stimulation, including the Mem‐ATP6V1A/Mem‐ATP1A1 ratio (F) and representative blots of membrane vs total V‐ATPase components (ATP1A1, ATP6V1A, and ATP6V0D1) (G). H. Schematic of the mechanism whereby sNPS mitigates endo/lysosomal acidification and restores endosomal trafficking balance, thereby dampening inflammatory outputs downstream of TLR4/LPS–NF‐κB and TLR3/poly(I:C)–IRF3. I. GO enrichment analysis of differentially expressed genes comparing sNPS vs PBS in the ischemic hemisphere. J. Representative flow cytometry plots and quantification of microglial polarization markers CD86 and CD206 in the ischemic ipsilateral hemisphere at 1, 3, and 7 days after PBS, SCS or sNPS administration. K. Representative immunofluorescence images showing microglia–neuron spatial association in the ischemic core at 1, 3, and 7 days, with quantification of MAP‐2^+^ microglia (cells mm^−2^). Data are presented as mean ± SD. *p < 0.05, **p < 0.01, ***p < 0.001, ****p < 0.0001.

We next examined whether this change was translated into suppression of downstream innate immune signaling. LPS increased the p‐p65/p65 ratio and reduced IκBα expression, whereas poly(I:C) increased the p‐IRF3/IRF3 ratio, confirming activation of the TLR4/NF‐κB‐ and TLR3/IRF3‐related pathways, respectively (Figure 5C; Figure S4G). SCS attenuated both signaling responses, while sNPS produced a more pronounced inhibitory effect that was comparable to pharmacological inhibition of endosomal acidification by BFA1 under the current experimental conditions. These findings indicate that nanoscale assembly enhanced the ability of SCS to suppress TLR3/4‐linked inflammatory signaling.

Inflammatory stimulation has been reported to enhance endosomal maturation, lysosomal engagement, and the associated acid‐loading burden in activated microglia [53]. Consistent with this response, LPS decreased Rab5‐associated fluorescence while increasing Rab7 and LAMP1 signals (Figure 5D,E), suggesting a shift away from the early endosomal state and toward enhanced late endosome/lysosome engagement under inflammatory stress. SCS partially attenuated these changes, whereas sNPS more effectively restored Rab5‐associated signals and reduced Rab7 and LAMP1 fluorescence. In parallel, LPS significantly increased the Mem‐ATP6V1A/Mem‐ATP1A1 ratio, indicating enhanced membrane recruitment of the V1A subunit of V‐ATPase (Figure 5F,G). Both SCS and sNPS reduced this increase, with sNPS showing the stronger inhibitory effect. In contrast, supplementary quantification showed no significant differences in Mem‐ATP6V0D1/Mem‐ATP1A1, total ATP6V0D1, or total ATP6V1A among groups (Figure S4H), indicating that the major effect of sNPS was not on overall V‐ATPase abundance, but rather on the membrane redistribution of ATP6V1A. Supporting transcriptional analyses further showed reduced iNOS expression together with increased anti‐inflammatory and neurotrophic‐factor‐related genes after sNPS treatment (Figure S4I). Collectively, these findings support a mechanism in which sNPS alleviates inflammation‐associated endo/lysosomal remodeling and limits V‐ATPase membrane recruitment, thereby reducing endo/lysosomal acid stress and downstream inflammatory signaling (Figure 5H).

To assess broader changes in the post‐ischemic immune microenvironment, RNA‐seq was performed using whole ischemic hemisphere tissue after PBS or sNPS treatment (Figure S5A–D). Venn analysis showed that, relative to the PBS group, sNPS treatment was associated with the loss of 1,288 expressed genes and the appearance of 1,066 additional genes. Furthermore, volcano‐plot analysis showed that the sNPS group contained 111 significantly upregulated genes and 144 significantly downregulated genes compared with the PBS group. GO enrichment analysis highlighted transcriptional changes related to interferon responses, innate immune regulation, defense responses, and MyD88‐dependent Toll‐like receptor signaling (Figure 5I). Consistently, representative genes involved in these processes showed altered expression after sNPS treatment (Figure S5D). Together, these transcriptomic data indicate that sNPS treatment is associated with broad changes in immune‐ and inflammation‐related transcriptional programs within the ischemic hemisphere.

We next examined whether these tissue‐level transcriptomic changes were accompanied by altered microglial phenotype in vivo. Flow cytometry showed that PBS‐treated mice maintained a relatively high proportion of CD86^+^ microglia during the early post‐ischemic stage. SCS produced a moderate reduction in the CD86^+^ population, whereas sNPS showed a more pronounced decrease, particularly on day 1 and day 3 (Figure 5J). In parallel, the CD86^−^/CD206^−^ population was increased after sNPS treatment, while SCS showed a weaker effect. These results indicate that sNPS primarily restrained the acquisition of a CD86‐associated pro‐inflammatory phenotype in vivo.

As microglia become activated, they undergo marked morphological remodeling and may abnormally engulf surviving neuronal components after ischemic stroke. We therefore examined whether sNPS could attenuate these in vivo changes in microglial morphology and microglia‐neuron interaction. By comparing different regions of the lesion, microglia were found to gradually accumulate from the penumbra toward the infarct core after stroke. In the ischemic core of PBS‐treated mice, microglial process retraction was already evident on day 1, and at later time points these cells exhibited a more activated morphology with shortened processes and increased association with MAP‐2^+^ neuronal material. SCS partially attenuated these morphological changes, whereas sNPS more effectively preserved ramified microglial morphology and reduced the association between Iba‐1^+^ microglia and MAP‐2^+^ neuronal structures during the early stage after stroke (Figure 5K; Figure S5G).

As previously reported, the pathological process after ischemic stroke can be divided into the hyperacute phase (minutes to 6 h), the acute and subacute phases (hours to 7 d), and the chronic phase (7 d to months) [54]. Consistent with this temporal framework, quantitative analysis showed that sNPS significantly reduced MAP‐2^+^ microglia on day 1 and day 3, whereas the difference was no longer significant by day 7 (Figure 5K), indicating that the effect of sNPS was most evident during the acute and subacute stages. Supplementary morphological analyses further supported this trend. In the ischemic core, PBS‐treated microglia displayed shortened processes and increased branch retraction, whereas sNPS‐treated microglia showed longer maximum and average branch lengths with a more ramified morphology. SCS produced moderate improvements in these morphological parameters but was generally less effective than sNPS (Figure S5E,F). Similarly, in the penumbra, sNPS also preserved longer and more highly branched microglial processes, while SCS showed a partial effect (Figure S5H‐I). Taken together, these results indicate that the endosomal pH‐regulating effect of sNPS is accompanied in vivo by restrained microglial activation and reduced pathological interaction with surviving neuronal structures after ischemic stroke.

### 2.5 sNPS‐Guided Neural Stem Cells Regulation and Cortical Preservation

Microglia influence stem cell behavior by secreting key trophic and inflammatory factors that regulate proliferation, differentiation, and survival [55]. Given that sNPS suppressed TLR3/TLR4‐related inflammatory signaling in microglia, we next asked whether this effect could be translated into a more favorable secretory profile for neural stem cell (NSC) regulation. ELISA showed that LPS increased the secretion of TNF‐α, IL‐6, IFN‐β, and IL‐10. CHQ and sNPS reduced TNF‐α, IL‐6, and IFN‐β secretion while further increasing IL‐10 levels (Figure S6A). The LPS‐induced increase in IL‐10 may reflect an endogenous compensatory response to inflammatory stimulation, whereas its further elevation after CHQ or sNPS treatment, together with the reduction in pro‐inflammatory cytokines, suggests a shift toward a more regulatory cytokine profile. These changes prompted us to examine downstream NSC responses in a Transwell coculture model (Figure 6A).

**FIGURE 6:**
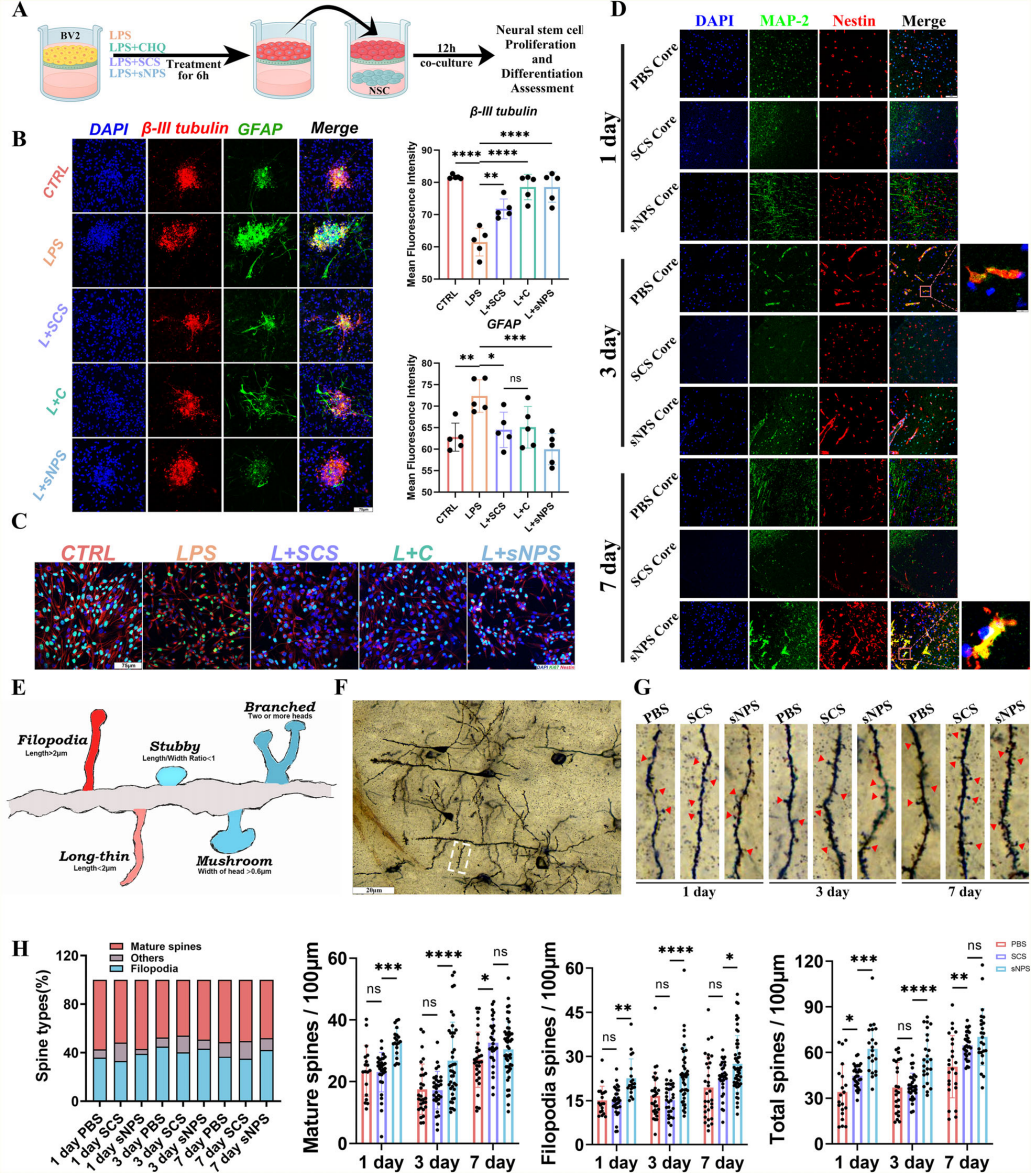
sNPS‐mediated microglial modulation is associated with NSC‐related responses and cortical structural preservation. A. Schematic of in vitro experiments investigating microglia‐mediated effects of sNPS on NSC fate. B. Representative images and quantitative analysis of β‐III tubulin and GFAP expression in NSCs following stimulation with differently treated BV2 cells. Scale bars were 75 µm. Data are shown as mean ± SD (n = 5). C. Representative images of proliferating NSCs following stimulation with differently treated BV2 cells. Scale bars were 75 µm. D. Representative immunofluorescence images showing MAP‐2^+^ neuronal structures and Nestin^+^ cells in the ischemic core of tMCAO mice at 1, 3, and 7 days after PBS, SCS, or sNPS administration. Scale bars: 50 µm (overview) and 5 µm (magnified images). E. Schematic diagram of different morphological types of dendritic spines. F‐G. Representative Golgi staining images of neurons in the cortex of tMCAO mice at low magnification (F). Scale bar = 20 µm. The white box indicates the region of secondary dendritic spines shown at higher magnification in (G). Different types of dendritic spines (red arrows) in the cortex of tMCAO mice at 1, 3 and 7 days post‐injection of sNPS, SCS or PBS are indicated in (G). H. Bar graph and quantification showing the proportion of each spine type, the number of filopodia spines (including filopodia and long‐thin spines), the number of mature spines (including stubby, mushroom, and branched spines), and the total number of spines in the infarct core region of tMCAO mice at 1, 3 and 7 days post‐treatment with sNPS, SCS or PBS. For each group at each time point, 30–50 randomly selected secondary dendritic spines were analyzed. *p < 0.05, **p < 0.01, ***p < 0.001, ****p < 0.0001.

Immunofluorescence staining first confirmed that the isolated NSCs retained stemness and exhibited the capacity to differentiate into both neuronal and glial lineages under unstimulated conditions (Figure S6B). Under inflammatory conditions, microglial conditioned medium increased GFAP and reduced β‐III tubulin expression in NSCs, consistent with previous reports [56]. SCS partially alleviated this differentiation imbalance, whereas CHQ and sNPS more clearly reduced GFAP fluorescence and restored β‐III tubulin expression. Among these treatments, sNPS showed a more pronounced restorative trend toward neuronal differentiation than SCS (Figure 6B). In parallel, Ki67/Nestin staining showed that LPS‐preconditioned microglia markedly suppressed NSC proliferative activity and reduced Nestin fluorescence. SCS produced a moderate recovery, whereas CHQ and sNPS better preserved Ki67^+^/Nestin^+^ cells and Nestin expression, with sNPS showing the strongest effect among the treatment groups (Figure 6C; Figure S6C).

Since excessive activation of microglia often results in structural and functional disruption of the cortex, leading to behavioral dysfunction, we next examined surviving neurons and the distribution of NSC‐related cells in the core and penumbra of the infarcted hemisphere. In vivo, sNPS better preserved MAP‐2‐associated neuronal structures in the ischemic core and was associated with time‐dependent changes in Nestin^+^/MAP‐2^+^ cell profiles in both the core and penumbra, whereas SCS generally produced weaker effects (Figure 6D; Figure S6D–F). Whole‐brain analysis was consistent with these regional observations, showing better preservation of cortical MAP‐2‐associated structures after sNPS treatment (Figure S6G). Consistent with the histological observations, sNPS partially restored NeuN and PSD‐95 expression, whereas changes in Synapsin‐I and Synaptophysin were less pronounced (Figure S6H‐I).

To investigate whether sNPS also influences dendritic spine remodeling after ischemic stroke, Golgi staining was employed to visualize neuronal dendritic spines. Based on their morphological characteristics, spines were classified into five categories: filopodia, long‐thin, stubby, mushroom, and branched spines (Figure 6E) [36]. Quantitative analysis of secondary dendrites showed that SCS produced partial but less pronounced changes in mature, filopodia, and total spine densities, whereas sNPS exerted a stronger and more consistent effect. Specifically, sNPS increased the total spine number at all observed time points and showed its most prominent preservation of mature spines during the early post‐stroke stage. In parallel, the number of filopodia spines was progressively increased by sNPS, whereas SCS did not produce a significant change in filopodia spine density (Figure 6F–H). These temporal changes suggest that sNPS was associated with early preservation of mature spine structures and subsequent dendritic spine remodeling and structural plasticity.

Taken together, these findings show that sNPS‐mediated modulation of microglial inflammatory responses was accompanied by improved NSC‐related marker profiles, neuronal structural preservation, and time‐dependent dendritic spine remodeling after ischemic stroke.

### 2.6 The Cerebral Protection of sNPS in the Acute and Subacute Phases of Stroke

After demonstrating the immunoregulatory and neuroprotective effects of sNPS in vivo, the overall therapeutic effects were further evaluated in mice following sNPS intervention. According to the experimental timeline, mice received retro‐orbital administration on day ‐1 and day 0, followed by histological analysis, behavioral assessment, and survival monitoring at the indicated time points (Figure 7A). Through reduction by dehydrogenase enzymes present in viable tissues to yield red‐colored formazan, 2,3,5‐triphenyltetrazolium chloride (TTC) was used to quantify cerebral infarction on day 1, 3, and 7 after PBS, SCS, or sNPS administration (Figure 7B). The sNPS group exhibited significantly lower infarct volume than the PBS group on day 1 and day 7, whereas SCS showed a partial reduction of infarct area (Figure 7C).

**FIGURE 7:**
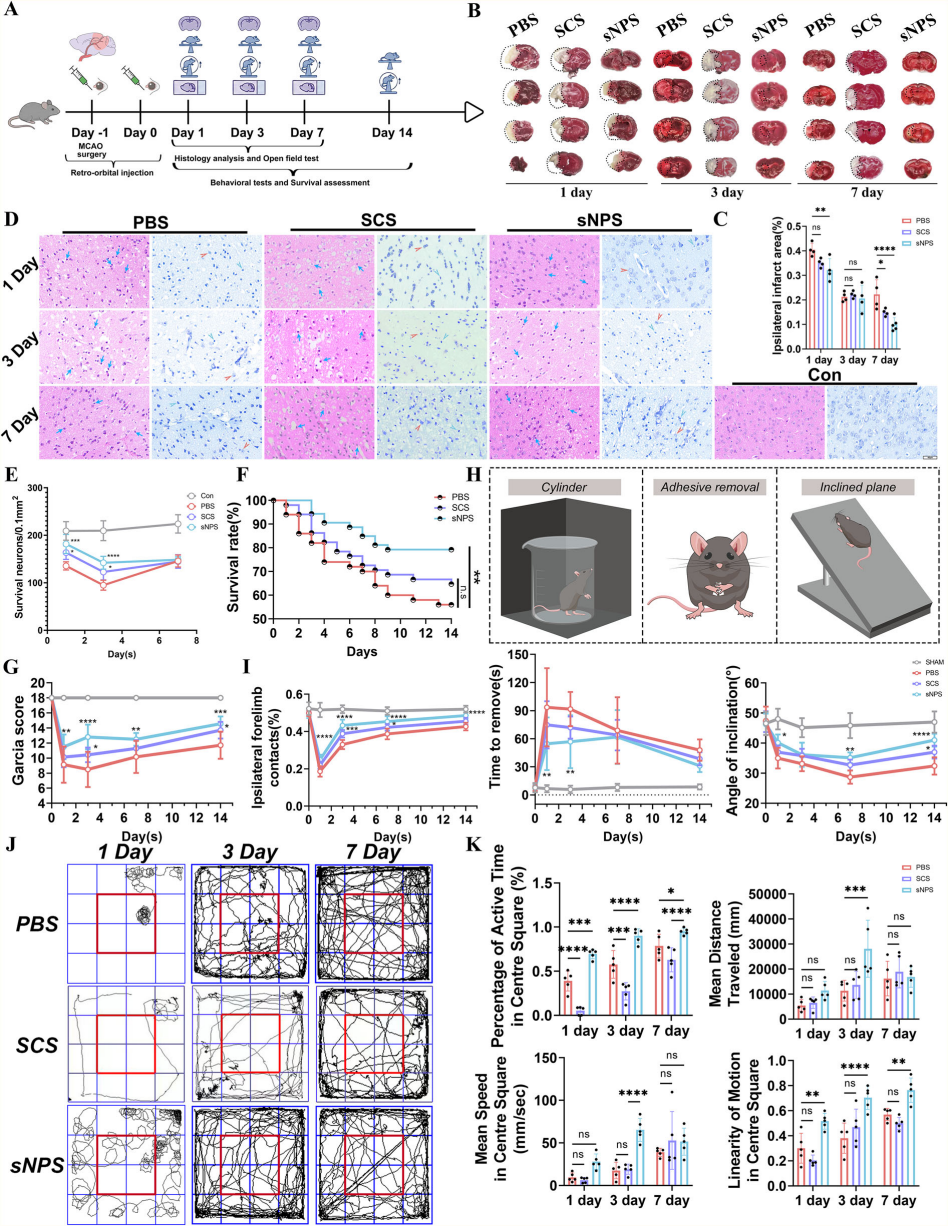
sNPS ameliorates the neurobehavioral functions and the histological damage caused by ischemia‐reperfusion. A. Schematic illustration of the experimental timeline for tMCAO surgery, retro‐orbital administration, histological analysis, behavioral assessments, and survival monitoring. B‐C. Representative TTC staining images (B) and quantitative results (C) of brain tissues from tMCAO mice at 1, 3 and 7 days post‐administration with sNPS (18 mg kg^−1^), SCS (18 mg kg^−1^) or PBS (100 µL) over two consecutive days. Data were shown as mean ± SD (n = 4–5). D‐E. Representative H&E and Nissl staining images of cortex from tMCAO mice at 1, 3 and 7 days post‐injection (D), along with quantitative results (E) of survival neuronal cells based on Nissl staining. Data were shown as mean ± SD (n = 5). Blue arrows in the H&E images: Cytosolic degradation; red arrows and blue arrows in the Nissl staining images: the survival neurons and apoptotic neurons. Con: ischemia contralateral hemisphere. Scale bar was 40 µm. F. Survival curves of tMCAO mice treated with PBS, SCS, or sNPS during the 14‐day observation period. (n = 50) G. Garcia scores of tMCAO mice at the indicated time points after PBS, SCS, or sNPS treatment. H–I. Schematic illustrations of the cylinder test, adhesive removal test, and inclined plane test (H), together with quantitative analysis of ipsilateral forelimb contacts, adhesive tape removal time, and tolerated inclination angle at the indicated time points (I). Data are presented as mean ± SD (n = 5–7). J‐K. Representative track plots of tMCAO mice at 1, 3 and 7 days post‐injection with sNPS, SCS or PBS during a 15‐min open field test (J) along with quantitative results for mean distance traveled, mean speed, linearity of motion, and percentage of active time in the center square (K). Data were shown as mean ± SD (n = 5). *p < 0.05, **p < 0.01, ***p < 0.001, ****p < 0.0001.

Cytoplasmic lysis and neuronal cell death at the infarction site of stroke were assessed by hematoxylin and eosin (H&E) staining and Nissl staining (Figure 7D). The H&E staining indicated massive cytosolic degradation (indicated by blue arrows) and tissue vacuoles in the infarct cortex in PBS group, which also explains the infarct tissue collapse observed in TTC staining. SCS partially ameliorated these pathological changes, whereas sNPS showed a more evident protective effect. Through the Nissl staining, neurotoxicity due to cerebral ischemia resulted in the transformation of normal neuronal cells (indicated by red arrows) into apoptotic neuronal cells (indicated by blue arrows), while quantification of surviving neuronal cells also showed that sNPS could rescue neurons from damage on the day 1 and 3, but not obvious on the day 7, indicating that the intervention effect of sNPS on mice after stroke was mainly concentrated in the acute and subacute stages of stroke (Figure 7E).

Survival analysis was then performed over a 14‐day observation period after tMCAO. Compared with PBS‐treated mice, sNPS administration improved the survival rate after stroke, while SCS showed an intermediate trend between PBS and sNPS (Figure 7F). In order to further determine the effect of sNPS on neurological function after ischemic stroke, the Garcia score was assessed at different time points according to the criteria summarized in Table S1. After tMCAO, PBS‐treated mice showed obvious neurological deficits, whereas SCS partially improved the Garcia score and sNPS produced a more pronounced recovery. The improvement in the sNPS group was maintained until day 14 (Figure 7G).

A series of additional neurobehavioral assessments were further performed to evaluate motor and sensorimotor function, including the cylinder test, adhesive removal test, and inclined plane test (Figure 7H). In agreement with the Garcia score, sNPS improved ipsilateral forelimb use in the cylinder test and increased the tolerated angle in the inclined plane test. A modest reduction in adhesive tape removal time was also observed, although this effect was less consistent across the assessed time points (Figure 7I).

Open‐field analysis showed outcome‐ and time‐dependent improvements after sNPS treatment, with the most evident changes observed in center‐zone activity and movement‐related parameters at the indicated post‐stroke time points. In contrast, SCS did not produce a consistent improvement (Figure 7J,K). It should be noted that PBS‐treated mice also exhibited a degree of spontaneous functional improvement over time, and the effects of sNPS therefore represent additional therapeutic benefits beyond this endogenous recovery process. Together, these results suggest that sNPS effectively alleviated cerebral injury and improved neuronal survival, motor coordination, and spontaneous exploratory behavior after ischemic stroke, with the most prominent benefits observed during the acute and subacute stages.

## 3 Conclusion

Current reperfusion and neuroprotective therapies for ischemic stroke remain limited by the complex and dynamic pathological microenvironment after ischemia‐reperfusion [57, 58], highlighting the urgent need for alternative interventions. Although numerous anti‐inflammatory drug‐loaded nanoparticles have been developed [50], research on the effects of bioactive materials on the ischemic stroke pathological process remains limited. Previous studies showed that surface‐charge‐engineered polypeptide‐gold nanoparticles could attenuate monocyte inflammatory responses through endosome‐mediated signaling pathways [59, 60], providing an important conceptual basis for considering endosomes as materials‐accessible regulators of immune‐cell behavior. Inspired by this finding, we investigated whether modulation of endosomal acidity could regulate microglial inflammatory responses after ischemic stroke. Pharmacological inhibition of endosomal acidification by BFA1 or CHQ attenuated pro‐inflammatory microglial responses, abnormal engulfment of neuronal material, and microglia‐associated neuronal apoptosis, supporting endosomal pH as a potential intracellular target. To mimic the effects of endosomal acidification inhibitors while avoiding their potential cytotoxicity, a material‐based strategy was established on a validated buffering scaffold. Structure‐function analysis of sulfonated chitosan derivatives indicated that its buffering behavior arises from the combined contributions of fixed sulfonate anions and titratable amines. Building on this basis, sNPS was constructed by nanoscale assembly to (i) increase the effective interfacial exposure of sulfonate and amino functionalities for endosomal pH regulation and (ii) enhance ischemic brain accumulation by converting SCS from a random‐coil polymer into nanoparticles with more favorable in vivo distribution and retention in the ischemic microenvironment. The resulting sNPS generally exerted stronger effects than unassembled SCS on endo/lysosomal acid stress, inflammation‐associated endosomal remodeling, V‐ATPase V1A membrane recruitment, and TLR4/NF‐κB‐ and TLR3/IRF3‐related signaling, accompanied by attenuation of maladaptive microglial responses after ischemic stroke.

To avoid overinterpretation of the multimodal evidence, the scope and limitations of the transcriptomic and phenotypic analyses should be explicitly clarified. The whole‐hemisphere RNA‐seq analysis should be interpreted as a tissue‐level assessment of transcriptional changes associated with sNPS treatment rather than as evidence of microglia‐intrinsic transcriptional reprogramming. The observed alterations in interferon‐related, innate immune, and MyD88‐dependent TLR signaling programs may reflect responses across multiple brain‐resident and infiltrating cell populations, as well as changes in cellular composition, immune‐cell infiltration, or injury severity. Therefore, the microglia‐related conclusions are supported primarily by the mechanistic studies in primary microglia together with in vivo flow cytometric, morphological, and microglia‐neuron association analyses. Future studies using sorted microglia or single‐cell RNA sequencing will be required to resolve cell‐type‐specific transcriptional responses. The CD86/CD206 analysis should likewise be interpreted within the limitations of this marker system, which provides only a coarse representation of post‐stroke microglial states. sNPS reduced the CD86^+^ fraction and increased the CD86^−^/CD206^−^ population, supporting attenuation of CD86‐associated inflammatory activation. Accordingly, the CD86/CD206 results were considered together with the complementary mechanistic and morphological evidence rather than as stand‐alone evidence of microglial reprogramming.

Post‐stroke inflammation and peripheral leukocyte recruitment have been exploited to facilitate nanoparticle delivery to ischemic lesions, with neutrophils and macrophages serving as potential cellular carriers [52]. Consistent with this concept, sNPS exhibited greater ipsilateral enrichment than SCS and was prominently associated with infiltrating F4/80^+^ macrophages, Ly6G^+^ neutrophils, and brain‐resident microglia. In the coculture model, sNPS‐associated FITC fluorescence and donor‐cell‐associated DiI signals were detected in cells morphologically identified as BV2 microglia, supporting the acquisition and redistribution of sNPS‐associated material during myeloid cell‐microglia interactions. However, these observations do not distinguish direct transfer of intact sNPS from uptake of donor‐cell‐derived membrane material, cellular fragments, or debris, and redistribution of the DiI label cannot be excluded. Moreover, BBB disruption, increased vascular permeability, passive extravasation, altered tissue retention, and residual vascular signals may contribute in parallel to ipsilateral enrichment. Consistently, FITC‐sNPS showed low leakage across the normal in vitro BBB model, whereas OGD injury progressively increased its passage. Thus, the present findings support a multifactorial model of ischemia‐associated sNPS enrichment rather than an exclusively immune‐cell‐mediated or BBB‐disruption‐dependent transport mechanism.

Another mechanistic consideration is the finite effective buffering reserve of sNPS. Its proton‐buffering activity depends on a limited number of interfacial sulfonate‐ and amine‐associated sites and is therefore concentration dependent rather than unlimited (Figure 3L). If sNPS resides in acidic endo/lysosomal compartments of peripheral neutrophils or macrophages before interacting with microglia, prior protonation may partially occupy its available buffering sites. Because protonation is reversible, subsequent exposure to less acidic extracellular or intracellular environments may restore part of this capacity. The residual buffering activity delivered to microglia is therefore likely to depend on the route and duration of cellular trafficking, local pH history, and particle retention or recycling. As this residual capacity was not directly quantified, future studies using pH‐history‐sensitive probes or sequential cell‐transfer models will be required to define the effective intracellular buffering lifetime of sNPS. Despite the potential constraints on proton‐buffering capacity discussed above, the in vivo therapeutic outcomes indicate that sNPS still produced measurable benefits after ischemic stroke. At the therapeutic level, sNPS reduced infarct injury and improved histological preservation after tMCAO. Improvements in survival and Garcia neurological scores remained evident during the 14‐day observation period, indicating that the therapeutic effects were not confined entirely to the early acute stage. Sensorimotor outcomes improved in an endpoint‐ and time‐dependent manner, with more evident effects in the cylinder and inclined plane tests and a comparatively modest and less consistent reduction in adhesive tape removal time. Open‐field analysis likewise showed time‐dependent improvements in spontaneous exploratory behavior, whereas SCS produced partial or inconsistent effects across the assessed therapeutic outcomes (Figure 7B–K). Together, these findings demonstrate therapeutic benefits across multiple histological and functional endpoints, although the magnitude and duration of improvement varied among individual outcomes.

Microglia can influence the post‐stroke neural niche through secreted inflammatory and trophic factors that regulate NSC behavior. In the present study, sNPS‐associated changes in the microglial cytokine profile were accompanied by partial preservation of NSC proliferative activity and differentiation‐associated marker profiles in the Transwell coculture model. Because ischemic injury evolves differently in the irreversibly damaged infarct core and the potentially recoverable penumbra [61], regional neural responses were further evaluated in vivo. sNPS treatment was associated with better preservation of MAP‐2‐associated neuronal structures and time‐dependent changes in Nestin^+^/MAP‐2^+^ cell profiles, whereas SCS generally showed weaker effects. Dendritic spine analysis further showed increased total spine density, early preservation of mature spines, and a progressive increase in filopodia‐associated spines. As filopodia serve as dynamic precursors involved in dendritic spine formation and structural plasticity [62]. These temporal changes suggest that sNPS supports both early preservation of existing synaptic structures and subsequent structural remodeling. Collectively, these findings support that sNPS‐mediated regulation of microglial responses contributes to a more favorable neural microenvironment, enhanced NSC‐related repair responses, and preservation and remodeling of neuronal structures after ischemic stroke.

In summary, this study establishes endosomal pH homeostasis as a materials‐accessible regulator of maladaptive microglial responses after ischemic stroke. Guided by the dual‐site proton‐buffering mechanism of SCS, sNPS were engineered through nanoscale assembly to integrate intrinsic pH‐regulating activity with improved ischemia‐associated brain enrichment. Following microglial uptake, sNPS alleviated endo/lysosomal acid stress, limited inflammation‐associated V‐ATPase membrane recruitment, and attenuated TLR3/4‐linked inflammatory signaling, thereby restraining maladaptive microglial activation. These effects were accompanied by improved neural repair‐related responses, neuronal structural preservation, survival, and functional outcomes. More broadly, these findings highlight a previously underexplored materials‐based strategy in which nanoscale engineering of a proton‐buffering interface—rather than cargo delivery alone—can shape microglial behavior and tissue repair, offering a complementary direction for therapeutic development in ischemic stroke.

## 4 Experimental Section/Methods

Detailed materials and methods are provided in SI Appendix, Materials and Methods, including the materials; Synthesis of sulfonated chitosan (SCS) derivative panel and sulfonated Nano Proton Scavengers (sNPS); Quantification of free primary amines by TNBS assay; Critical Micelle Concentration (CMC) Measurement; Acid‐base titration; preparation and characterization of FITC‐labeled SCS and sNPS; primary microglia isolation; immunofluorescence analysis of IL‐6 and Arg‐1 expression in BV2 cells; endosomal acidification inhibitors‐mediated inflammation suppression in vitro; NSC isolation and microglial modulation of stem cell fate; RNA analysis by qRT‐PCR; western blotting; assessment of endosomal acidification; transmission electron microscopy analysis of primary microglia; endosomal maturation marker staining in primary microglia; transfer of sNPS from neutrophils/Raw264.7 to BV2; mouse transient middle cerebral artery occlusion (tMCAO) model; biodistribution of sNPS in tMCAO mice; in vitro Transwell BBB permeability assay; assessment of cerebral infarction volume by TTC staining; histology and immunofluorescence; Golgi‐Cox staining; flow cytometry analysis; behavioral tests; RNA‐seq and data analysis; statistical analysis. All surgical procedures were approved by the Institutional Animal Care and Use Committee of East China University of Science and Technology.

## Author Contributions

**Zehua Gao**: conceptualization, methodology, investigation, validation, Writing – review and editing. **Xuanlin Wang**: conceptualization, methodology, investigation, validation, supervision, visualization, writing – original draft, writing – review and editing, project administration. **Yujing Shi**: investigation, methodology, validation, writing – review and editing, conceptualization. **Jing Wang**: conceptualization, supervision, project administration, funding acquisition, writing – review and editing. **Changsheng Liu**: conceptualization, project administration, supervision, funding acquisition.

## Funding

This work was supported by the Key Program of the National Natural Science Foundation of China (No. 32230059), the Basic Science Center Program of the National Natural Science Foundation of China (No. T2288102), the National Natural Science Foundation of China (No. 32471406), the Foundation of Frontiers Science Center for Materiobiology and Dynamic Chemistry (No. JKVD1211002), and the Shandong Province Key Research and Development Project (No. 2023CXPT103).

## Conflicts of Interest

The authors declare no conflicts of interest.

## Supporting information




**Supporting File**: advs77311‐sup‐0001‐SuppMat.doc.

## Data Availability

The data that support the findings of this study are available from the corresponding author upon reasonable request.
