# Supplementary material for: Nano Proton Scavengers Modulate Endosomal pH to Inhibit Microglial Activation and Enhance Stroke Recovery
Source: Adv Sci (Weinh). 2026 Aug 21;13(62):e77311. doi: 10.1002/advs.77311 (PMC13496290; doi:10.1002/advs.77311)
Supplement: Supplementary file 1 [file ADVS-13-e77311-s001.doc]

**Supporting Information**

**Materials and methods**

**Materials**

Chitosan with a molecular weight of 3.0 × 10^5^ and a deacetylation degree of 92% was purchased from Shenzhen Zhongfayuan Biological Technology Co., Ltd. (Shenzhen, China). Chlorosulfonic acid was purchased from Sinopharm Chemical Reagent Co., Ltd. (Shanghai, China). Oleic acid was purchased from Naicheng Biotechnology Co., Ltd. (Shanghai, China). DMF, formamide, formic acid, pyrene and EDC were purchased from Yien Chemical Technology Co., Ltd. (Shanghai, China). 2% Triphenyltetrazolium chloride solution was purchased from Solarbio Science & technology Co., Ltd. (Beijing, China). Flow cytometry antibodies were purchased from Dakewei Biotechnology Co., Ltd. (Shenzhen, China). Immunofluorescent antibodies were purchased from Abcam (St. Louis, MO, United States). Western blotting antibodies were purchased from Cell Signaling Technology, Inc. (Boston, MA, United States). All cell culture related reagents were purchased from Gibco (Grand Island, NY, United States). CCK-8 cell proliferation and viability detection kits were purchased from Beyotime Biotechnology Co., Ltd. (Shanghai, China).

**Synthesis of sulfonated chitosan (SCS) derivative panel and sulfonated Nano Proton Scavengers (sNPS)**

SCS was synthesized as previously reported.^[1]^ In brief, 5 mL HClSO₃ was added dropwise to 50 mL DMF in an ice bath to prepare the sulfonating reagent for SCS-HS. For SCS-LS, the sulfonating reagent was prepared identically except that the HClSO₃ concentration was reduced by half (i.e., 2.5 mL HClSO₃ in 50 mL DMF), while all other reaction conditions were kept the same. Chitosan (2.5 g) was added to 50 mL formamide containing 4% (v/v) formic acid, and after being fully dissolved, the sulfonating reagent was added drop by drop. The reaction was carried out at 50°C under argon protection for 2 h. Absolute ethanol was added to precipitate SCS. After pH adjustment and dialysis against deionized water for three days, the products were obtained by lyophilization. FTIR analysis was performed using a Fourier transform infrared spectrometer (Nicolet 380, Thermo, USA) to demonstrate the successful grafting of sulfonate groups.

PEG-modified sulfonated chitosan (SP) was prepared by EDC/NHS-mediated amide coupling between SCS-HS and carboxyl-terminated PEG (PEG2000–COOH). Briefly, SCS-HS was dissolved in 50 mL DMF. PEG2000–COOH was then added at molar ratios (primary amines on SCS-HS : PEG2000–COOH) of 1:1, 1:2, 1:4, and 1:8, respectively. Subsequently, EDC and NHS (1.5 equiv each, relative to PEG2000–COOH) were added, and the reaction was allowed to proceed at room temperature for 24 h under stirring. The reaction mixture was purified by dialysis against deionized water using a 12 kDa MWCO dialysis membrane for 3 days with frequent water changes. The purified products (SP series) were obtained by lyophilization. Successful PEG2000 conjugation was further confirmed by aqueous gel permeation chromatography (GPC), using a water-based mobile phase, with molecular weight calibration performed using water-soluble standards.

Arginine-modified sulfonated chitosan (ASCS) was synthesized via EDC/NHS-mediated amide coupling using SCS-HS as the backbone. Briefly, SCS-HS was dissolved in DMF to form a homogeneous solution. L-arginine (Arg) was then added at a molar ratio of Arg : free primary amines on SCS-HS = 1:1. Subsequently, EDC and NHS (1.5 equiv each, relative to Arg) were added, and the reaction mixture was stirred at room temperature for 24 h. The resulting product was purified by dialysis against deionized water using a 12 kDa MWCO dialysis membrane for 3 days with frequent water changes, followed by lyophilization to obtain ASCS.

Arginine-modified chitosan (ACS) was synthesized via EDC/NHS-mediated amide coupling in MES buffer. Chitosan (CS) was dissolved in MES buffer (25 mM, pH 5.5), and L-arginine (Arg) was introduced at a molar ratio of CS repeating units : Arg = 1:1. In parallel, Arg was activated in MES buffer by sequential addition of EDC and NHS at 1.5 equivalents each (relative to Arg), followed by stirring in an ice-water bath for 2 h. The activated Arg solution was then added to the CS solution, and the coupling reaction was allowed to proceed at 30°C for 24 h. The reaction was quenched by adjusting the pH to 8.0 using 1.0 M NaOH. The product was purified by dialysis against distilled water using a 3.5 kDa MWCO dialysis membrane for 4 days, followed by lyophilization to obtain ACS as an off-white flocculent solid. Successful arginine grafting was further confirmed by ^1^H NMR (solvent: D_2_O), which showed the appearance of characteristic arginine resonances in ACS compared with pristine chitosan.

Because of the hydrophilicity of sulfonate groups, hydrophobic oleic acid was grafted to the sulfonated chitosan molecular chain to form amphiphilic nanoparticles. SCS-HS and oleic acid were dissolved at molar ratios ranging from 1:1000 to 1:5000 in 1 mL of phosphate buffer solution and 150 mL of DMF containing 2% (w/v) EDC. Subsequently, the oleic acid-EDC mixture was added dropwise to the SCS solution and the reaction went on for 24 hours. After being dialyzed against deionized water for three days and lyophilized, sNPS were prepared. Infrared spectra of sNPS were measured. The particle size, zeta potential and isoelectric point were measured through the dynamic light scattering particle size analyzers (Zetasizer Nano ZS, Malvern Panalytical). The surface morphology of the nanoparticles was observed by transmission electron microscopy (JEM-2100, JEOL).

**Quantification of free primary amines by TNBS assay**

The content of free primary amines was quantified using the 2,4,6-trinitrobenzenesulfonic acid (TNBS) assay under alkaline conditions. Briefly, 125 μL of sample solution was mixed with 1.0 mL of PBS buffer (pH 8.2) and 1.0 mL of 0.1% (w/v) TNBS solution. The mixture was incubated at 50°C for 1 h in the dark with shaking to allow derivatization. The reaction was then quenched by adding 2.0 mL of 1% (w/v) NaHSO₃, followed by incubation in the dark for an additional 30 min. Absorbance was measured at 420 nm using a UV–Vis spectrophotometer/microplate reader. A reagent blank (PBS + TNBS processed identically without sample) was used for background subtraction. A standard curve was generated using L-leucine solutions at known concentrations prepared in the same PBS buffer, and the free primary amine content was calculated from the calibration curve and reported as the equivalent amine concentration (mM).

**Critical Micelle Concentration (CMC) Measurement**

The critical micelle concentration (CMC) of sNPS was determined by fluorescence spectroscopy using pyrene as a hydrophobic fluorescent probe. A methanol solution of pyrene (6.0 × 10^−5^ M) was added to a 6-well plate, and the methanol was evaporated using a nitrogen gas stream. A series of sNPS solutions with concentrations ranging from 2.5 to 5000 μg mL^-1^ were prepared and equilibrated with a constant concentration of pyrene (6.0 × 10^−7^ M) at 37°C for 24 hours. Under an excitation of 337 nm, the intensity ratio of the first peak (I_1_ at 373 nm) to the third peak (I_3_ at 383 nm) was plotted against the logarithm of polymer concentration. The CMC value was determined by identifying the intersection of the tangent to the curve at the inflection point with the horizontal tangent line.

**Acid-base titration**

Acid–base titration was performed to evaluate the buffering behavior of SCS derivatives and sNPS under different titration settings. Titrations were conducted by stepwise addition of 0.01 M HCl with continuous pH monitoring until the pH reached 4.0, and titration curves were plotted as the cumulative HCl volume added versus the corresponding pH value. For the comparison among different chitosan derivatives in dH_2_O (Figure 3D), SCS-HS, SCS-LS, ASCS, ACS, the SP series, and sNPS were each dispersed in dH₂O at 5 mg mL^-1^, and titrated directly with 0.01 M HCl under the conditions described above. To examine the influence of different starting pH values on buffering performance (Figure 3K), sNPS solutions (5 mg mL^-1^) were first adjusted to the desired initial pH using 0.01 M NaOH or 0.01 M HCl as appropriate. After equilibration, the solutions were titrated with 0.01 M HCl to pH 4.0 with continuous pH recording as described above. To assess buffering behavior in protein-containing medium (Figure 3L), 5 mg mL^-1^ sNPS, 5 μg mL^-1^ sNPS, and 5 mg mL^-1^ SCS were dissolved and re-suspended in deionized water and 10% FBS, respectively. The solutions in deionized water and 10% FBS were adjusted to the same titration starting point. All solutions (5 mL) were gradually titrated to pH 4.0 with 0.01 M HCl.

**Preparation and characterization of FITC-labeled SCS and sNPS**

FITC-labeled SCS (FITC-SCS) and sNPS (FITC-sNPS) were prepared through the reaction between FITC and the residual amino groups of SCS or sNPS. Briefly, SCS or sNPS was dispersed in sodium bicarbonate buffer (0.5 M, pH=9.6), followed by the addition of FITC. The reaction was performed in the aqueous phase at room temperature under gentle stirring in the dark for 24 h. After reaction, the samples were dialyzed against deionized water for 5 days using dialysis membranes with a molecular-weight cutoff of 10 kDa. The dialysis medium was replaced regularly, and the FITC concentration in the dialysate was measured using a microplate reader at excitation and emission wavelengths of 488 and 525 nm, respectively. FITC concentrations were calculated using a standard calibration curve. The purified FITC-SCS and FITC-sNPS were collected and stored at 4 ℃ in the dark.

FITC labeling efficiency was calculated as:

$$FITC labeling efficiency(\%)=\frac{m_{0}-m_{free}}{m_{0}}\times100$$

where m_0_ represents the initial amount of FITC added and m_free_ represents the cumulative amount of free FITC detected in the dialysate.

To evaluate whether FITC labeling affected nanoparticle properties, the hydrodynamic diameter and zeta potential of sNPS and FITC-sNPS were measured.

For fluorescence-labeling stability, FITC-SCS and FITC-sNPS were incubated in PBS containing 20% serum at 37 °C in the dark. At predetermined time points, aliquots were collected and transferred into centrifugal ultrafiltration tubes with a molecular-weight cutoff of 10 kDa. After centrifugation at 14000×g for 15 min, released FITC in the filtrate was quantified using a microplate reader at excitation and emission wavelengths of 488 and 525 nm, respectively. FITC retention was calculated as follows:

$$FITC retention(\%)=\left( 1-\frac{m_{released}}{m_{total}} \right)\times100$$

where m_released_ represents the amount of released FITC detected in the filtrate and m_total_ represents the total amount of FITC associated with the labeled material before incubation.

**Primary microglia isolation**

Primary microglia were isolated from fetal mice as previously reported.^[2]^ In brief, after removal of the meningeal lining, the whole brains from eight fetal BALB/c mice were collected and minced into pieces. The brain pieces were further dissociated with trypsin/EDTA (0.05%/0.02%) (Gibco, Cat:25300120) for 10 min. After neutralization with 10% DMEM (Gibco, Cat:11965092), the supernatant was filtered through a 70 μm cell strainer, and the resuspended cells were cultured with 15% DMEM. When the cells were approximately 80-90% confluent, the microglia were detached from the flask using the heated orbital shaker at 240 rpm for 2 h (37 °C). The cell purity was assessed via flow cytometry with Cytoflex LX (Beckman Coulter, United States) and immunofluorescence analysis with a confocal microscope (Leica, Germany).

**Immunofluorescence analysis of IL-6 and Arg-1 expression in BV2 cells**

To assess the expression of pro-inflammatory and anti-inflammatory factors in microglia following inhibition of endosomal acidification, immunofluorescence was employed. After treatment with LPS (1 μg mL^-1^), LPS + Bafilomycin A1 (1 μM) (MedChemExpress, United States) and LPS + chloroquine (20 μM) (MedChemExpress, United States) for 3 hours, microglial cell line BV2 cells (Pricella Biotechnology Procell Co., Ltd., Wuhan) were fixed with 4% neutral paraformaldehyde buffer for 15 minutes and permeabilized with 0.3% Triton X-100 for 15 minutes. The samples were then stained overnight at 4°C with primary antibodies against IL-6 (Cell Signaling Technology, 12912T) and Arg-1 (Cell Signaling Technology, 93668T), followed by staining with fluorescently labeled secondary antibodies at room temperature for 1 hour. The stained samples were subsequently examined using a laser confocal microscope (Leica, TCS SP8).

**Endosomal Acidification Inhibitor-mediated Inflammation Suppression *in vitro***

To evaluate the effects of endosomal acidification inhibitors on abnormal phagocytosis in microglia after LPS stimulation, a co-culture model of BV2 and HT22 cells was established. BV2 cells were seeded at a density of 5 × 10^4^ cells per confocal dish (NEST, Cat:801001) and cultured for 24 hours. The culture medium was then replaced with glucose-free medium containing LPS (1 μg mL^-1^), LPS + Bafilomycin A1 (1 μM), or LPS + chloroquine (20 μM). After treatment for 6 hours, HT22 cells were seeded at a density of 2 × 10^4^ cells per confocal dish. Co-cultures were maintained for an additional 1 hour or 3 hours, after which the cells were fixed for further staining. Samples were analyzed using laser confocal microscopy, and the number of BV2 cells expressing MAP-2 mm^-^² was quantified.

The rescue effect of inhibiting endosomal acidification on HT22 cell apoptosis induced by pro-inflammatory microglia was assessed using the Annexin-V FITC/PI assay. Briefly, HT22 cells were seeded at a density of 2.7 × 10^4^ cells per well in the basal chamber of a 24-well plate and incubated overnight. The medium was then replaced with glucose-free medium. BV2 cells were seeded at a density of 1 × 10^4^ cells per well in the apical chamber. After different treatments were applied for 3 hours, the medium was replaced with glucose-free medium, and the BV2 cells were co-cultured with HT22 cells in a transwell system for an additional 3 hours. Subsequently, flow cytometry was used to quantify the proportions of early and late apoptosis in HT22 cells.

**NSC Isolation and Microglial Modulation of Stem Cell Fate**

NSCs were isolated from the cerebral cortices of E13.5–E14.5 C57BL/6 mouse embryos. Dissected cortical tissues were enzymatically dissociated using 0.25% trypsin-EDTA at 37 °C for 10 minutes, followed by mechanical trituration. The resulting single-cell suspension was filtered (70 μm), centrifuged, and resuspended in serum-free NSC medium (Knockout DMEM/F12 (Gibco, Cat:12660012) plus StemPro Neural Supplement (Gibco, Cat:A1050801), 20 ng mL^-1^ EGF and 20 ng mL^-1^ b-FGF). Cells were cultured in uncoated flasks at 37 °C in 5% CO₂, and neurospheres were collected after 3–5 days.

To assess how sNPS influences neural stem cell (NSC) proliferation and differentiation via microglial modulation, a transwell co-culture system was employed. Briefly, NSCs were seeded at a density of 5 × 10^6^ cells per well in the lower chamber of a 24-well plate and incubated overnight. BV2 microglial cells were seeded at a density of 1 × 10^4^ cells per insert in the upper chamber. After different treatments were applied for 6 hours, the medium was replaced with glucose-free medium, and BV2 cells were co-cultured with NSCs for an additional 12 hours. After co-culture, cells were fixed with 4% paraformaldehyde and subjected to subsequent immunofluorescence staining procedures. Immunofluorescence staining was performed as previously described. Primary antibodies used included anti-GFAP (Cell Signaling Technology, 3670T), anti-β-III tubulin (Cell Signaling Technology, 5568T), anti-Nestin (Cell Signaling Technology, 89529S), and anti-Ki67 (Cell Signaling Technology, 9129T).

**RNA analysis by qRT-PCR**

The effect of endosomal acidity on the expression of inflammation-related genes in the BV2 cells under an LPS-simulated inflammatory environment was investigated using qRT-PCR. BV2 cells were treated with LPS (1 μg mL^-1^), LPS + Bafilomycin A1 (1 μM), and LPS + chloroquine (20 μM) for 3 hours, after which the total RNA was extracted.

To explore the impact of sNPS on the expression of inflammation-related and neurotrophic factor-related genes in microglia after glucose and oxygen deprivation (OGD), qRT-PCR was performed. To simulate the hypoxic environment following ischemic stroke in vitro, OGD treatment was conducted as previously reported.^[3]^ Briefly, isolated microglia were rinsed with HBSS and incubated in a hypoxic chamber (Thermo Fisher Scientific, Waltham, Massachusetts, USA) filled with a gas mixture of 5% CO_2_, 3% O_2_ and 92% N_2_ for 4 hours. After OGD treatment, the HBSS was replaced with DMEM complete medium (containing 10% FBS) supplemented with sNPS at concentrations of 1 μg mL^-1^, 2 μg mL^-1^, 4 μg mL^-1^, 8 μg mL^-1^ and 12 μg mL^-1^, and the cells were further incubated for 12 hours before RNA extraction.

Total RNA was extracted from the microglia using Trizol reagent (Takara, Cat:9109), and reverse transcription to cDNA was performed using the PrimeScript RT reagent kit (Takara, Cat:RR047A) according to the manufacturer's guidelines. qRT-PCR was conducted using the CFX96 Touch PCR detection system (Bio-Rad, United States). Primer sequences are listed in Table S2. All results were normalized, and all samples were measured in triplicate.

**Western blotting**

Primary microglia were seeded in 6-well plates and subjected to inflammatory stimulation followed by the indicated interventions. For the TLR4 model, cells were treated with LPS (1 μg mL^-1^), LPS + SCS (12 μg mL^-1^), LPS + sNPS (12 μg mL^-1^), LPS + bafilomycin A1 (BFA1, 1 μM), or LPS + sNPS (12 μg mL^-1^) + BFA1 (1 μM) for 12 h. For the TLR3 model, cells were treated with poly(I:C) (50 μg mL^-1^), poly(I:C) + SCS (12 μg mL^-1^), poly(I:C) + sNPS (12 μg mL^-1^), poly(I:C) + BFA1 (1 μM), or poly(I:C) + sNPS (12 μg mL^-1^) + BFA1 (1 μM) for 6 h. After treatment, cells were lysed in RIPA buffer containing PMSF (Beyotime, Shanghai, China) at 4°C for 30 min. Lysates were clarified by centrifugation, and total protein concentrations were determined using a BCA assay kit (Beyotime, Cat:P0010). Equal amounts of protein were separated by SDS–PAGE and transferred to 0.22 μm PVDF membranes (Millipore, USA). Membranes were blocked with blocking buffer (Beyotime, Cat:P0252) at room temperature for 30 min and incubated with primary antibodies at 4°C overnight. After incubation with HRP-conjugated secondary antibodies, signals were visualized using a chemiluminescence imaging system (Tanon, Shanghai, China). Band intensities were quantified and normalized to β-actin as the loading control.

For membrane-associated protein analysis, microglia were treated with LPS (1 μg mL^-1^), LPS + SCS (12 μg mL^-1^), or LPS + sNPS (12 μg mL^-1^) for 12 h. Membrane and cytosolic fractions were extracted using the Membrane and Cytosol Protein Extraction Kit (YEASEN, Shanghai, China; Cat. 20127ES50) according to the manufacturer’s instructions. Membrane fractions and total lysates were subsequently analyzed by western blotting as described above. Membrane-associated targets were normalized to ATP1A1 for membrane recruitment quantification (e.g., Mem-ATP6V1A/Mem-ATP1A1), while total protein bands were normalized to β-actin.

For neuronal and synaptic protein analysis, the ischemic hemisphere was collected from mice treated with PBS or sNPS (18 mg kg⁻¹). Tissues were homogenized and lysed in RIPA buffer containing PMSF on ice, and protein quantification and immunoblotting were performed as described above. Band intensities were quantified and normalized to β-actin. The suppliers and catalog numbers of all antibodies used are provided in Table S3.

**Assessment of endosomal acidification**

To determine the endosomal acidification process and dextran uptake in microglia, cells treated with LPS (1 μg mL^-1^), LPS + SCS (12 μg mL^-1^), LPS + sNPS (12 μg mL^-1^), LPS + bafilomycin A1 (BFA1, 1 μM), or LPS + sNPS (12 μg mL^-1^) + BFA1 (1 μM) for 12 h were incubated with pHrodo Red dextran (10 μg mL^-1^) (Thermo Fisher Scientific, Cat:P35368) and FITC-labeled dextran (30 μg mL^-1^) (Thermo Fisher Scientific, Cat:D7178) for 20 min, then fixed with 4% paraformaldehyde for 30 min, washed with PBS, and imaged by laser confocal microscopy. Fluorescence intensities of pHrodo Red and FITC signals were quantified using ImageJ.

For unfixed-cell validation, primary microglia were subjected to the same treatment and pHrodo-dextran loading procedures described above. After probe incubation, cells were washed with prewarmed PBS and immediately imaged without paraformaldehyde fixation under identical acquisition settings.

**Transmission electron microscopy analysis of primary microglia**

Primary microglia were treated with sNPS (12 μg mL^-1^) for 12 h, while untreated cells served as the control. After treatment, the cells were gently washed with pre-cooled 0.1 M phosphate buffer (pH 7.4) and initially fixed in situ with 2.5% glutaraldehyde for 15 min. The cells were then gently collected using a cell scraper, centrifuged at 1000×g for 5 min, and further fixed in fresh 2.5% glutaraldehyde at 4℃ for 2 h. After three washes with phosphate buffer, the cell pellets were post-fixed with 1% osmium tetroxide for 1 h and dehydrated through a graded ethanol series. Ultrathin sections with a thickness of 90 nm were prepared, collected on copper grids, and sequentially stained with uranyl acetate and lead citrate. Cellular ultrastructure was examined using a Hitachi HT7800 transmission electron microscope. Endo/lysosomal morphology, including vesicular swelling, limiting-membrane integrity, and leakage-like ultrastructural changes, was evaluated in randomly selected cells.

**Endosomal maturation marker staining in primary microglia**

To assess inflammation-associated changes in endo/lysosomal trafficking, primary microglia were treated with LPS, LPS + FITC-SCS (12 μg mL^-1^) or LPS + FITC-sNPS (12 μg mL^-1^) for 6 h. Cells were then fixed with 4% paraformaldehyde for 15 min, washed with PBS, permeabilized with 0.3% Triton X-100 for 15 min, and blocked with 5% goat serum for 1 h at room temperature. Subsequently, cells were incubated overnight at 4°C with primary antibodies against Rab5 (Cell signaling technology, Cat:3547), Rab7 (Cell signaling technology, Cat:9367), and LAMP1 (Cell signaling technology, Cat:99437). After washing, cells were incubated with the corresponding fluorescently labeled secondary antibodies for 1 h at room temperature. Nuclei were counterstained with DAPI, and images were acquired using a laser confocal microscope (Leica TCS SP8) under identical imaging settings across groups. Mean fluorescence intensity (MFI) of Rab5, Rab7, and LAMP1 signals was quantified using ImageJ, with background subtraction applied consistently across all images.

**Coculture of sNPS-loaded neutrophils or RAW264.7 macrophages with BV2 microglia**

Primary mouse neutrophils were isolated as previously described.^[4]^ Neutrophils and the mouse macrophage cell line Raw264.7 (Pricella Biotechnology Procell Co., Ltd., Wuhan) were stained with DiI, a cell membrane marker, for 30 minutes. Excess DiI was then thoroughly washed off with PBS before the experiment. FITC-sNPS was co-cultured with neutrophils or Raw264.7 for 12 hours, after which non-internalized sNPS was thoroughly removed. Neutrophils or Raw264.7 at a concentration of 5 × 10^4^ cells mL^-1^ were then added to the mouse microglial cell line BV2 at the same concentration. After 6 h of coculture, the distributions of FITC-sNPS-associated and DiI-associated fluorescence were examined using laser scanning confocal microscopy.

**Mouse transient middle cerebral artery occlusion (tMCAO) model**

Male C57BL/6 mice were purchased from JieSiJie Laboratory Animal Co., Ltd. (Shanghai, China). All procedures were approved by the Animal Research Committee of East China University of Science and Technology. The ethical approval number was ECUST-2024-050. A mouse tMCAO model was constructed by monofilament method.^[5]^ Briefly, mice were anesthetized and placed on the warming pad to maintain body temperature. In tMCAO mice, the left carotid artery, internal carotid artery and external carotid artery were separated and exposed. The external carotid artery was ligated and a small cut was made. The suture (0.10 mm) wrapped with a silicone tip (0.20 to 0.22 mm, RWD, Shanghai, China) was inserted from the opening of the external carotid artery, followed by the internal carotid artery until resistance was met (occluding the middle cerebral artery). After 1 hour embolization, the suture was removed and the wound was sutured. Mice in the sham group underwent the same process of anesthesia and common carotid artery exposure, but without the MCAO procedure. The success of occlusion was confirmed by monitoring the blood flow of middle cerebral artery branches (CBF) with Laser Speckle Contrast Imaging (RFLSI III, RWD, Shanghai China). After tMCAO treatment, retro-orbital injections of PBS, 18 mg kg^-1^ SCS or 18 mg kg^-1^ sNPS were given for two consecutive days.

**Biodistribution of sNPS and SCS in tMCAO mice**

tMCAO mice were injected with FITC-conjugated SCS or sNPS (18 mg kg^-1^) via retro-orbit to evaluate their biodistribution. 6, 12 and 24 h after administration, mice were sacrificed and major organs were extracted. The fluorescence images of organs were observed on IVIS Lumina III (Perkin Elmer, Hopkinton, MA, USA) and the fluorescence signals were overlaid with the brightfield images.

**In vitro Transwell BBB permeability assay**

An in vitro BBB coculture model was established using bEnd.3 mouse brain endothelial cells and C8-D1A astrocytes in 24-well Transwell inserts (Corning, Cat. No. 3415). bEnd.3 cells and C8-D1A astrocytes were co-seeded onto the apical surface of the Transwell membrane at a density of 5 × 10⁴ cells cm^-2^ for each cell type to establish a direct-contact endothelial–astrocyte coculture. Barrier formation was monitored on days 1, 3, 5, and 7 using a 10 kDa FITC-dextran permeability assay. Briefly, FITC-dextran (0.5 mg mL⁻¹) was added to the apical chamber and incubated at 37 °C for 1 h. Medium from the basolateral chamber was subsequently collected, and fluorescence intensity was measured. FITC-dextran permeability was calculated as permeability (%) = (F_lower/F_initial input) × 100%, where F_lower represents the fluorescence intensity of the basolateral sample and F_initial input represents the fluorescence intensity of the FITC-dextran solution initially added to the apical chamber under identical measurement settings. FITC-dextran permeability was used as a functional indicator of barrier formation. Based on the stabilization of FITC-dextran permeability by day 5, a 5-day coculture period was used for the subsequent normal and OGD-injured BBB permeability experiments. After formation of the barrier model, the normal BBB group was maintained under standard culture conditions, whereas the injured BBB groups were subjected to oxygen-glucose deprivation (OGD) for 2, 4, or 6 h. FITC-sNPS was then added to the apical chamber, and medium from the basolateral chamber was collected after the indicated incubation period. Fluorescence intensity was measured and the FITC-sNPS leakage rate was calculated as: leakage rate (%) = (F_lower / F_initial input ) × 100%, where F_lower represents the fluorescence intensity of the basolateral sample and F_initial input represents the fluorescence intensity of the FITC-sNPS solution initially added to the apical chamber under the same measurement settings. All groups were analyzed under identical fluorescence measurement settings.

**Assessment of cerebral infarction volume by TTC staining**

2, 3, 5-triphenyltetrazolium chloride (TTC) staining was performed to evaluate infarction volume in the tMCAO model. At 1, 3, 7 days after retro-orbital injection for two consecutive days, the mice were euthanized; the brains were carefully extracted and sliced into 2 mm coronal sections. The slices were incubated in 2% TTC solution at 37°C for 20 minutes, protected from light. After fixation in 4% paraformaldehyde for 30 minutes, ImageJ was used to evaluate the infarction volume of each slice. The total infarction volume was obtained by the summing of each slice.

**Histology and immunofluorescence**

The whole brain of mice was collected, fixed in 4% neutral paraformaldehyde buffer for 4 h, dehydrated, and embedded in paraffin or OCT (Leica, Germany) for sectioning. For histological analysis, sections of hearts, kidneys, lungs, spleens, livers and brains were stained with hematoxylin and eosin (H&E) to observe the inflammatory cell infiltration and cytoplasmic lysis. The brain sections were further stained with Nissl staining solution (Wuhan servicebio technology CO., Ltd., Cat:G1036) to observe the survival neurons. For immunofluorescence, the sections were permeabilized with 0.3% Triton X-100 for 15 min, and then blocked with 5% goat serum for 1 hour. The samples were stained with primary antibodies Iba-1 (Abcam, ab178847); F4/80 (Abcam, ab90247); Ly6G (Abcam, ab25377); NeuN (Abcam, ab177487); GFAP (Abcam, ab68428); Nestin (Abcam, ab221660) and MAP-2 (Abcam, ab302487) overnight at 4℃, followed by incubation with fluorescence labelled secondary antibodies for 1 hour at room temperature. Nuclei were stained with DAPI (Cell signaling technology, Cat:8961) and the images were captured by Laser confocal microscope (Leica, TCS SP8).

**Golgi-Cox staining**

For Golgi-Cox staining, the manufacturer’s instructions (Golgi-cox staining experiment report, Wuhan servicebio technology CO., Ltd.) were strictly followed. tMCAO mice were deeply anesthetized prior to euthanasia. The entire brains were promptly extracted from the skull, quickly rinsed with PBS to remove surface blood and immediately placed into Golgi stain fixing solution. After 3 days of fixation, the stain fixing solution was replaced with Golgi dye solution. Tissue blocks were stored in 26℃, away from light, for 2 weeks. Post-staining, the dye solution was discarded and the tissue treatment solution was replaced for 3 days at 4℃. The tissues were then sliced into 60 μm sections and the tissue treatment solution was applied to the slices, which were allowed to dry slightly before development. The slices were rinsed with dH_2_O and developed using Golgi developer solution for 30 minutes. Multi-planes images were acquired under bright field using the Pannoramic 250 FLASH (3DHISTECH). Subsequent measurements were conducted using ImageJ software.

**Flow cytometry analysis**

Flow cytometry was performed to evaluate the proportions and cellular distribution of FITC-SCS- and FITC-sNPS-associated cells in the ischemia ipsilateral and contralateral hemispheres, as well as the activation and polarization of microglia. To the former, FITC-conjugated sNPS and SCS (18 mg kg^-1^) were injected into tMCAO mice via retro-orbit. After 12 and 24 h, the ipsilateral and contralateral hemisphere of ischemia was extracted and digested with 0.25% trypsin (Thermofisher, Cat:25200056) for 10 min at 37°C. To identify FITC^+^ cell types, cells were stained with BV510-conjugated Zombie viability dye (biolegend, 423101) to exclude dead cells, followed by surface staining with PE/Cy7-conjugated CD45 (biolegend, 368532), PE-conjugated CD11b (biolegend, 379903), PerCP/Cy5.5-conjugated Ly6G (biolegend, 127615), and BV421-conjugated F4/80 (biolegend, 123131). The proportion of FITC⁺ cells and their distribution among immune cell subsets were analyzed by flow cytometry. While the latter involved harvesting and mincing the brains from tMCAO mice, followed by digestion of the minced tissues with 0.25% trypsin for 10 minutes at 37°C with gentle agitation. After filtration through a 70 μm cell strainer, the cell suspension was washed with HBSS and re-suspended with flow cytometry staining buffer at a concentration of 1 × 10^6^ cells mL^-1^. 100 µL of cell suspension (1 × 10^5^ cells) was separated and stained with APC/Cy7-conjugated 7-AAD (Thermofisher, 00-6993-50), PE-conjugated CD11b (biolegend, 379903), PE/Cy7-conjugated CD45 (biolegend, 368532), APC-conjugated CD86 (biolegend, 374208) and FITC-conjugated CD206 (biolegend, 321104) for 30 min at 4℃. The cells were washed twice and analyzed with Cytoflex LX (Beckman coulter, United States). FlowJo_V10 was used to evaluate the cell ratio.

**Behavioral Tests**

*Garcia score:* An 18-point score developed by Garcia et al.^[6]^was used to assess the overall improvement in motor and coordination function in mice after the intervention of PBS, SCS or sNPS as presented in Table S1.

*Adhesive removal test:* The adhesive removal test was used to evaluate the coordination and sensitivity of forepaw movements. Tapes were applied to the ipsilateral and contralateral forepaws for assessment of perceptual and motor function after tMCAO. The time for mice to remove the tape from both limbs was recoreded over a period of 120 s. The mean value of three trials was presented. 1 day before tMCAO, the time spent to remove the tapes was measured as the preoperative data. Adhesive removal test was performed at 1, 3 and 7 days after continuous retro-orbital injections.

*Cylinder test:* The cylinder test was used to evaluate the preference of mice for the undamaged forepaw during exploration. After 1, 3 and 7 days continuous retro-orbital injections, mice were placed into a clean, open-top glass cylinder (diameter: 18 cm, height: 25cm) in a quiet room with dim lighting. The movement and exploration behavior of mice were recorded for 5 min. All scoring was performed by the experimenter blind to the condition of the mice. The number of ipsilateral, contralateral and bilateral touches to the cylinder wall were measured, and the data is presented as a percentage of the damaged forepaw relative to the total touches.

*Inclined plane test:* The inclined plane test is used to evaluate the improvement of forelimb strength in mice with PBS, SCS or sNPS intervention after ischemic stroke. After the mice were placed on the inclined plane which is raised in 5° increments, the maximum angle at which the mice maintained on the inclined plane for at least 5s was recorded as the inclined plane score. Each trial was repeated three times and the average angle was measured. The inclined plane test is performed at 1, 3 and 7 days after continuous retro-orbital injections for 2 days.

*Open field test:* Open field test was used to assess the autonomous movement and space exploration of mice. The mice were placed from the bottom left corner into a 40 cm$\times$40 cm blue plexiglass box and 15 min free exploration was allowed. Ethovision XT software was used to analyze mouse movement trajectories and various movement-related parameters were measured.

**RNA-seq and data analysis**

After two days of consecutive injections of sNPS or PBS, the ischemic hemispheres were carefully extracted and rapidly frozen using liquid nitrogen. The tissues were then lysed in Total RNA Extractor kit (B511311, Sangon, China) according to the manufacturer’s instructions, and genomic DNA was removed using RNase-free DNase I. Subsequent RNA sequencing and library preparation were performed by Sangon Biotech Co., Ltd. (Shanghai, China).

1 μg of RNA per sample was used for library preparation with the VAHTSTM mRNA-seq V2 Library Prep Kit for Illumina®. The library quality was evaluated using the Agilent Bioanalyzer 2100 system. FastQC (version 0.11.2) was used to assess the quality of the sequenced data. Trimmomatic (version 0.36) filtered raw reads by removing adaptor sequences, low-quality bases, and short reads. The remaining clean data were mapped to the reference genome using HISAT2 (version 2.0). Alignment results were statistically analyzed with RSeQC (version 2.6.1). Genome structure and homogeneity were checked using Qualimap (version 2.2.1), and gene coverage ratios were analyzed with BEDTools (version 2.26.0). Gene expression levels were calculated using StringTie (version 1.3.3b). TPM (Transcripts Per Million) normalized gene expression values. DESeq2 (version 1.12.4) identified differentially expressed genes (DEGs) with q-value ≤ 0.05 and |FoldChange| ≥ 2. Gene expression differences were visualized using heatmaps, venn plot and volcano plot. Gene Ontology (GO) enrichment analysis was used to identify significantly enriched terms among DEGs using a hypergeometric test.

**Statistical analysis**

The quantitative results were analyzed using GraphPad Prism software (version 9.0.0). For comparisons involving multiple experimental groups, one-way or two-way analysis of variance (ANOVA) was conducted. Survival curves were estimated using the Kaplan-Meier method and compared using the log-rank (Mantel-Cox) test. All statistical data are presented as mean ± standard deviation (SD).

Figure. S1. The Abnormal Phagocytosis of Microglial Cells. A. Representative immunofluorescence images of HT22 cells phagocytosed by BV2 cells after 3 hour co-culture in an inflammatory environment. Scale bar was 30 μm.

**Figure. S2. Derivative controls for elucidating the buffering origin of SCS and complementary characterization of sNPS. A**. Representative ^1^H NMR spectra of L-arginine (LA) and arginine-modified chitosan (ACS), with characteristic arginine-associated signals highlighted (red dashed boxes), confirming successful arginine incorporation. **B.** GPC profiles showing the molecular weight distributions of SCS and the PEG–SCS series (SP1–SP8) used as derivative controls. **C.** FTIR spectra of chitosan (CS) and sulfonated chitosan (SCS), with characteristic absorption bands associated with sulfonate groups indicated. **D.** FTIR spectra of SCS and sNPS, with characteristic peaks corresponding to oleic acid (OA) grafting (alkyl C–H stretching) marked. **E.** Representative TEM image of sNPS (scale bar: 500 nm)**. F.** Zeta potential of SCS and sNPS. **G.** Cell viability of BV2 cells treated with different concentrations of sNPS for 1 day and 3 days. Data are presented as mean ± SD (n = 5).

**Figure. S3. Verification of successful tMCAO modeling, FITC labeling, in vitro BBB barrier formation and permeability assessment, ischemia-associated hemispheric distribution of sNPS, and in vivo biocompatibility. A**. Representative laser speckle imaging of cortical regional cerebral blood flow (CBF) in male C57BL/6 mice (6–8 weeks), acquired before surgery, during MCAO, and after 1 h reperfusion, confirming effective occlusion and reperfusion in the tMCAO model. **B**. FITC concentration in the dialysate during dialysis of FITC-SCS and FITC-sNPS, showing the removal of unbound FITC. **C**. Hydrodynamic diameter and zeta potential of sNPS before and after FITC labeling. **D**. FITC labeling efficiency of SCS and sNPS and fluorescence retention of FITC-SCS and FITC-sNPS after incubation in 20% serum-containing medium. **E**. Quantification of FITC⁺ cell ratio in the contralateral hemisphere at 12 h and 24 h after administration, showing no significant differences between groups (ns). **F**. Ex vivo fluorescence imaging of brain slices from sham and tMCAO mice at 12 h and 24 h post-injection. **G**. Schematic illustration of the in vitro Transwell BBB coculture model. Barrier formation was evaluated by measuring the permeability of 10 kDa FITC-dextran on days 1, 3, 5, and 7 of coculture. The established barrier model was subsequently subjected to OGD for 2, 4, or 6 h, and FITC-sNPS leakage across the normal and OGD-injured barriers was quantified to evaluate the effect of ischemia-like barrier injury on sNPS passage. Data are presented as mean ± SD (n = 5). **H**. Representative H&E staining of major organs (liver, spleen, heart, lung, and kidney) at 48 h after treatment with 18 mg kg^-1^ sNPS in tMCAO mice. Scale bar: 100 μm. Data are presented as mean ± SD. ns, not significant; *P < 0.05, **P < 0.01, ***P < 0.001,****P < 0.0001.

**Figure. S4. Supporting evidence for sNPS-mediated modulation of primary microglia. A.** Representative immunofluorescence images of isolated primary microglia stained for Iba-1 (red) with nuclei counterstained by DAPI (blue), confirming microglial identity. Scale bar: 300 μm. **B.** Representative flow cytometry gating and histogram showing CD11b expression to evaluate the purity of the isolated primary microglia. **C.** Representative confocal images showing intracellular colocalization of pHrodo–dextran (green) and FITC-labeled sNPS (red) in primary microglia. Scale bar: 10 μm. **D-E.** Representative unfixed pHrodo-dextran images (**D**) and quantitative analysis of pHrodo fluorescence intensity (**E**) in primary microglia under the indicated treatments. **F.** Representative TEM images of primary microglia after control or sNPS treatment, showing cellular ultrastructure and endo/lysosomal compartments. **G.** Quantification of innate immune signaling proteins shown in Figure 5C, including p-p65, IκBα, and p-IRF3 expression under the indicated treatments. **H.** Supplementary quantification for Figure. 5G of membrane pump–related proteins in primary microglia, including the Mem-ATP6V0D1/Mem-ATP1A1 ratio and the expression of Total-ATP6V0D1 and Total-ATP6V1A under the indicated treatments. **I**. qRT–PCR analysis of gene expression in primary microglia following the indicated treatments, including inflammation-associated markers (iNOS, Tgf-β, Il-4, Il-10) and neurotrophic factors (BDNF, GDNF, NT3, NGF). Data are presented as mean ± SD (n = 3). *P < 0.05, **P < 0.01, ***P < 0.001, ****P < 0.0001.

**Figure. S5. Supplementary whole-hemisphere transcriptomic and microglial morphological analyses following sNPS treatment in the post-ischemic brain. A.** Experimental timeline for retro-orbital administration, RNA-seq, histological analysis, and flow cytometry after tMCAO. **B.** Venn diagram showing the overlap of expressed genes between the PBS and sNPS groups. **C.** Volcano plot of differentially expressed genes (DEGs) for sNPS vs. PBS, using the indicated thresholds (|log₂ fold change| > 1, adjusted P < 0.05). **D.** Heatmap of the 19 differentially expressed genes highlighted in the GO categories shown in Figure.5I. **E-F.** Quantification of microglial morphological parameters in the ischemic core at 1, 3, and 7 days after treatment, including maximum branch length (E), branch number, and average branch length (F). **G.** Representative immunofluorescence images in the penumbra showing microglia (Iba-1, red) and neurons (MAP-2, green) with nuclei counterstained by DAPI (blue) at 1, 3, and 7 days for the following conditions: contralateral hemisphere (Con), penumbra in PBS-treated mice (PBS), penumbra in SCS-treated mice (SCS) and penumbra in sNPS-treated mice (sNPS). Scale bar: 50 μm. **H-I.** Quantification of microglial morphology metrics corresponding to (**G**) across the indicated time points and conditions. Data are presented as mean ± SD. *P < 0.05, **P < 0.01, ***P < 0.001, ****P < 0.0001.

**Figure. S6. Supplementary immune- and neural-related assessments following sNPS treatment. A.** ELISA quantification of cytokines in microglia-conditioned medium under the indicated treatments, including TNF-α, IL-6, IFN-β, and IL-10. **B.** Representative immunofluorescence images of neurospheres derived from isolated neural stem cells (NSCs), stained for lineage-associated markers as indicated. Scale bars: 60 μm. **C.** Quantification of Ki67^+^/Nestin^+^ cells and Nestin fluorescence intensity in NSCs after indirect Transwell coculture with BV2 microglia subjected to the indicated treatments. Data are presented as mean ± SD (n = 5). **D.** Quantification of Nestin^+^/MAP-2^+^ cells in the ischemic core at 1, 3, and 7 days after PBS, SCS, or sNPS treatment. **E.** Quantification of Nestin^+^/MAP-2^+^ cells in the ischemic penumbra at the indicated time points. **F.** Representative immunofluorescence images in the penumbra showing MAP-2^+^ neurons (green) and Nestin^+^ cells (red) with nuclei counterstained by DAPI (blue) at 1, 3, and 7 days after PBS, SCS or sNPS administration; conditions are presented as contralateral hemisphere (PBS Con), penumbra in PBS-treated mice (PBS Penum), penumbra in SCS-treated mice (SCS Penum) and penumbra in sNPS-treated mice (sNPS Penum). Scale bar: 50 μm. **G.** Representative whole-brain immunofluorescence images for Nestin and MAP-2 at 1, 3, and 7 days post-injection of PBS, SCS or sNPS. **H–I.** Western blotting of neuronal /synaptic proteins in the ischemic hemisphere, with representative blots (**H**) and quantification (**I**), including NeuN, Synapsin-I, Synaptophysin, and PSD-95. *P < 0.05, **P < 0.01, ***P < 0.001, ****P < 0.0001.
**Table S1.** Garcia score^[6]^

| **Test(score)** | **3** | **2** | **1** | **0** |
| --- | --- | --- | --- | --- |
| **Spontancous activity (5min)** | At least 3 walls | 1~2 walls | Spin in circle | Stay still |
| **Physical symmetry** | Symmetrical movement | Contralateral slight movement | Contralateral hemiplegia | ... |
| **Forelimbs stretches** | Symmetrical outreach | Contralateral moves and outreaches less than ipsilateral side | Contralateral mild movement to outreach | Contralateral hemiplegia |
| **Griping and climbing cages** | Complete climbing and grasping ability | Contralateral injury performance | No climbing or spinning in circles | ... |
| **Bilateral body tactile reflex** | Symmetrical response | Contralateral weak response | Contralateral no response | ... |
| **Bilateral whiskers tactile reflex** | Symmetrical response | Contralateral weak response | Contralateral no response | ... |

**Table S2.** Primer sequences for qRT-PCR.

| **Gene** | **Primer sequences** |
| --- | --- |
| Gapdh | Forward:CGGAGTCAACGGATTTGGTCGTAT |
|  | Reverse:AGCCTTCTCCATGGTGGTGAAGAC |
| iNOS | Forward:GCAGAGATTGGAGGCCTTGTG |
|  | Reverse:GGGTTGTTGCTGAACTTCCAGTC |
| Tgf-β | Forward:CTCCCGTGGCTTCTAGTGC |
|  | Reverse:GCCTTAGTTTGGACAGGATCTG |
| Il-4 | Forward:AGATGGATGTGCCAAACGTCCTCA |
|  | Reverse:AATATGCGAAGCACCTTGGAAGCC |
| Il-10 | Forward:ATAGTCCTTCCTAGCCCAATTTCC |
|  | Reverse:GATGAATTGGATGGTCTTGGTCC |
| Il-1β | Forward:TCCAGGATGAGGACATGAGCAC |
|  | Reverse:GAACGTCACACACCAGCAGGTTA |
| Il-6 | Forward:CTGTACTGCAAAAACGGG |
|  | Reverse:AAAGTATAGCTTTCTGCC |
| Tnf-α | Forward:CAGGCGGTGCCTATGTCTC |
|  | Reverse:CGATCACCCCGAAGTTCAGTAG |
| Bdnf | Forward:GCGGCAGATAAAAAGACTGC |
|  | Reverse:GCAGCCTTCCTTGGTGTAAC |
| Gdnf | Forward:ATGAAGTTATGGGATGTCGTGGCTG |
|  | Reverse:ACCGTTTAGCGGAATGCTTTCTTAG |
| Nt3 | Forward:ACCGAACTCGAGTCCACCT |
|  | Reverse:TGGAATTCTGACCTGGTGGC |
| Ngf | Forward:ACACTCTGATCACTGCGTTTTTG |
|  | Reverse:CCTTCTGGGACATTGCTATCTGT |

**Table S3.** Primary antibodies used for western blotting

| **Target** | **Supplier** | **Catalog No.** |
| --- | --- | --- |
| Phospho-NF-κB p65 | Cell Signaling Technology | 3033T |
| NF-κB p65 | Cell Signaling Technology | 8242T |
| IκBα | Cell Signaling Technology | 2859T |
| β-actin | Cell Signaling Technology | 4970S |
| Phospho-IRF3 | Cell Signaling Technology | 79945T |
| IRF3 | Cell Signaling Technology | 4302T |
| ATP1A1(Na^+^/K^+^-ATPaseα1) | Abcam | Ab76020 |
| ATP6V1A | Cell Signaling Technology | 39517T |
| ATP6V0D1 | Abcam | Ab202899 |
| NeuN | Cell Signaling Technology | 24307T |
| PSD-95 | Cell Signaling Technology | 3450T |
| Synapsin-I | Cell Signaling Technology | 5297T |
| Synaptophysin | Cell Signaling Technology | 5461T |

**Reference**

[1] H. Zhou, J. Qian, J. Wang, et al. Enhanced bioactivity of bone morphogenetic protein-2 with low dose of 2-N, 6-O-sulfated chitosan in vitro and in vivo, Biomaterials 30(9) (2009) 1715-1724.

[2] X. Chen, Y. Zhang, G. Sadadcharam, W. Cui, J.H. Wang. Isolation, Purification, and Culture of Primary Murine Microglia Cells, Bio-protocol 3(1) (2013) e314.

[3] W. Xin, Y. Pan, W. Wei, et al. Preconditioned extracellular vesicles from hypoxic microglia reduce poststroke AQP4 depolarization, disturbed cerebrospinal fluid flow, astrogliosis, and neuroinflammation, Theranostics 13(12) (2023) 4197-4216.

[4] Z.H. Gao, Y.M. Yu, K. Dai, et al. Engineering Neutrophil Immunomodulatory Hydrogels Promoted Angiogenesis, ACS Applied Materials & Interfaces 14(35) (2022) 39746-39758.

[5] L. Belayev, O.F. Alonso, R. Busto, W. Zhao, M.D. Ginsberg. Middle cerebral artery occlusion in the rat by intraluminal suture. Neurological and pathological evaluation of an improved model, Stroke 27(9) (1996) 1616-1622; discussion 1623.

[6] J.H. Garcia, S. Wagner, K.F. Liu, X.J. Hu. Neurological deficit and extent of neuronal necrosis attributable to middle cerebral artery occlusion in rats. Statistical validation, Stroke 26(4) (1995) 627-634; discussion 635.
